# Early biomarkers of brain injury and cerebral hypo- and hyperoxia in the SafeBoosC II trial

**DOI:** 10.1371/journal.pone.0173440

**Published:** 2017-03-22

**Authors:** Anne M. Plomgaard, Thomas Alderliesten, Topun Austin, Frank van Bel, Manon Benders, Olivier Claris, Eugene Dempsey, Monica Fumagalli, Christian Gluud, Cornelia Hagmann, Simon Hyttel-Sorensen, Petra Lemmers, Wim van Oeveren, Adelina Pellicer, Tue H. Petersen, Gerhard Pichler, Per Winkel, Gorm Greisen

**Affiliations:** 1 Department of Neonatology, Rigshospitalet, Copenhagen University Hospital, Copenhagen, Denmark; 2 University Medical Center Utrecht, Wilhelmina Children’s Hospital, Utrecht, The Netherlands; 3 Rosie Hospital Cambridge University Hospitals NHS Foundation Trust, Cambridge, United Kingdom; 4 Department of Neonatology, Hospices Civils de Lyon, Claude Bernard University, Lyon, France; 5 INFANT Centre, University College Cork, Cork, Ireland; 6 NICU,Fondazione IRCCS Ca’ Granda Ospedale Maggiore Policlinico, Università degli Studi di Milano, Milan, Italy; 7 Copenhagen Trial Unit, Centre for Clinical Intervention Research, Rigshospitalet, Copenhagen University Hospital, Copenhagen, Denmark; 8 Clinic of Neonatology, University of Zurich, Zurich, Switzerland; 9 Haemoscan B.V., Groningen, The Netherlands; 10 Department of Neonatology, La Paz University Hospital, Madrid, Spain; 11 Research Unit on Brain Injury Neurorehabilitation Copenhagen, Department of Neurorehabilitation, TBI Unit, Rigshospitalet, Copenhagen University Hospital, Hvidovre, Denmark; 12 Department of Pediatrics, Research Unit for Neonatal Micro- and Macrocirculation, Medical University of Graz, Graz, Austria; Centre Hospitalier Universitaire Vaudois, FRANCE

## Abstract

**Background:**

The randomized clinical trial, SafeBoosC II, examined the effect of monitoring of cerebral oxygenation by near-infrared spectroscopy combined with a guideline on treatment when cerebral oxygenation was out of the target range. Data on cerebral oxygenation was collected in both the intervention and the control group. The primary outcome was the reduction in the burden of cerebral hypo- and hyperoxia between the two groups. In this study we describe the associations between the burden of cerebral hypo- and hyperoxia, regardless of allocation to intervention or control group, and the biomarkers of brain injury from birth till term equivalent age that was collected as secondary and explorative outcomes in the SafeBoosC II trial.

**Methods:**

Cerebral oxygenation was continuously monitored during the first 72h of life in 166 extremely preterm infants. Cranial ultrasound was performed at day 1,4,7,14, and 35 and at term. Electroencephalogram (EEG) was recorded at 64h. Blood-samples taken at 6 and 64 hours were analysed for the brain injury biomarkers; S100beta, brain-fatty-acid-binding-protein, and neuroketal. All analyses were conducted post hoc.

**Results:**

Significantly more infants with a cerebral burden of hypoxia within the 4^th^ quartile versus infants within quartile 1–3 were diagnosed with severe intracranial haemorrhage (11/39 versus 11/117, p = 0.003), had low burst rate on EEG (12/28 versus 21/103, p = 0.015), or died (14/41 versus 18/123, p = 0.006), whereas none of these events were significantly associated with cerebral hyperoxia. The blood biomarkers were not significantly associated with the burden of cerebral hypo- or hyperoxia.

**Conclusions:**

The explorative analysis showed that early burden of cerebral hypoxia, but not hyperoxia was significantly associated with low brain electrical activity and severe intracranial haemorrhage while none of the three blood biomarkers were associated with the burden of either cerebral hypo- or hyperoxia.

## Introduction

Extremely preterm infants have an immature cardiorespiratory system and cerebral autoregulation can be impaired, especially during the first days of life [1,2]. This makes the developing brain of the preterm infant susceptible to fluctuations in the cerebral blood flow (CBF) [3] and may cause episodes of cerebral hypo- and hyperoxia. Near-infrared spectroscopy (NIRS) is a non-invasive method for estimating tissue oxygenation. NIRS measures the ratio of the concentrations of oxygenated haemoglobin to total haemoglobin on an absolute scale with a range of 0% to 100% [4]. Changes in cerebral NIRS-values are correlated to CBF [5]. A number of biomarkers–characteristics that is objectively measured and evaluated as an indicator of normal biological processes, pathogenic processes, or pharmacologic responses to a therapeutic intervention [6]—may be present if the brain is suffering from hypo- or hyperoxia. Severe intraventricular haemorrhage (IVH grade III) and periventricular haemorrhagic infarction (PVHI) mainly develop within the first 3 days of life [7], during the period of transition from intra- to extra-uterine life when the brain is especially vulnerable. Low cerebral oxygenation, as estimated by NIRS, during this transition has been associated with higher grades of intraventricular haemorrhage and lower 2-year developmental quotients [8–10]. In addition high values of NIRS in animal studies are associated with brain injury [11], as confirmed in human asphyxiated term new-borns [12]. Cerebral NIRS monitoring is currently used in some neonatal intensive care units as part of the standard of care for extremely preterm infants and infants with hypoxic-ischemic encephalopathy. Yet it remains to be determined if monitoring cerebral oxygenation combined with clinical interventions when cerebral oxygenation levels are out of range actually prevents cerebral injury, improves neurological outcome, and/or increases the survival of the extremely preterm infants [13].

The phase II randomized clinical trial, SafeBoosC II, demonstrated that it is possible to reduce the burden of cerebral hypoxia during the first 72 hours of life [14]. The SafeBoosC II study was, however, not powered to detect differences in clinical outcomes [15]. In the present post hoc analysis, we use the SafeBoosC II data to explore the association between the burden of cerebral hypo- and hyperoxia regardless of trial allocation to intervention or control group, and the secondary and explorative outcomes of the trial (all being potential biomarkers of brain injury or death): namely serial cranial ultrasound (cUS), electroencephalographic (EEG) measures, and blood molecular markers.

## Patients and methods

### Infant characteristics and study design

SafeBoosC II is a multicentre randomised clinical feasibility trial [15]. A total of 166 extremely preterm infants were included in the SafeBoosC II study before 3 hours of age: 86 infants were randomised to the experimental group (cerebral NIRS monitoring in combination with an evidence based intervention guideline [16] for NIRS values out of range (55–85%)) and 80 infants to the control group (blinded collection of NIRS values combined with treatment as usual). The inclusion criteria were infants born more than 12 weeks before term (gestational age <27 weeks and six days) with a decision to provide full life support and the possibility to start cerebral NIRS monitoring within three hours after birth. Written informed consent from the parents was mandatory before inclusion and randomisation. The randomisation was web based and handled by The Copenhagen Trial Unit. The generated allocation sequence of 1:1 with block sizes 4 and 6 in random order concealed for the investigators. The intervention period was 72 hours. The 2y-followup of the infants is on going. The infants were recruited from 8 European countries each represented by one neonatal intensive care unit (June 2012 to December 2013). The trial is registered at ClinicalTrial.gov, NCT01590316, the protocol is available in full at http://www.safeboosc.eu.

### The burden of cerebral hypo- and hyperoxia

The primary outcome of the SafeBoosC II trial was the burden of hypo- and hyperoxia. This was calculated as the time spent below or above the target limits multiplied by the mean deviation from the lower (55%) or the upper limit (85%) during the first 72 hours of life, expressed in percentage hours (%hours). The burden was computed from un-edited NIRS-values and extrapolated to 72 hours, without knowledge of any other outcomes of the trial [14].

### Cranial ultrasound

On day 1 (anytime during the first 24 hours of life), 4 (± 1), 7 (± 1), 14 (± 1), and 35 (± 1) and at term equivalent age (week 38 to 44) standardized cUS (6 coronal and 5 sagittal images through the anterior fontanel and one through the mastoid window) was performed. The images were anonymised and uploaded to a central server. The images were centrally analysed by two experts (CH and MB) using the software program OsiriX version 6.0 (Pixmeo, Geneva, Switzerland). The process of the central scoring is described in detail elsewhere [17]. IVH grade III, PVHI, post haemorrhagic ventricular dilatation, porencephalic cysts, cystic periventricular leukomalacia, cerebral atrophy at term, stroke and cerebellar haemorrhage at one or more of the scans were classified as severe brain injury, and thereby as severe adverse outcome.

### Electroencephalogram

EEG was analysed in 133 infants, the median age at EEG-recording was 65 hours postnatal and the median time of recording was 2 hours [18]. Electrodes were placed at P3 and P4 position according to the international 10-20-system. Needle, disc or hydrogel electrodes were used according to local practice. The electrode impedance was less than 20kΩ during the recording. If the child was treated with morphine, other opioids, or sedative medications this was documented.

All EEG analysis was performed in Matlab version R2014b using custom build programmes (MathWorks, Natick, Massachusetts, USA), without knowledge of the medical history of the infant. The analysis of the EEGs is described in detail elsewhere [18].

The raw EEG was band pass filtered (0.5–30 Hz) using a zero phase filter and converted into range-EEG (rEEG) [19]. Artefacts in the rEEG were independently visually identified by two of the authors (GG and AMP) blinded to the clinical history of the infant. The EEG analysis was conducted on the remaining artefact-free data.

Burst rate was calculated as the number of bursts per minute. A burst was defined as nested (high frequency) oscillations within large slow-wave depolarisations using an extraction algorithm based on the co-occurrence of a slow (0.5–2 Hz) wave and higher (8–22 Hz) frequency oscillation, as described by Hartley et al. [20]. Consecutive events occurring within 0.5 seconds of one another were counted as one and events of duration less than 4/22 of a second were discounted [20]. Burst rates were significantly affected (decreased burst rate) by the use of morphine and EEG-recordings with an online filtration at 2–15 Hz (decreased burst rate) [18]. Therefore the burst rates were adjusted for these variables. Adjusted burst rate within the 1^st^ quartile was considered an adverse outcome.

Spectral-analyses were conducted using Matlab routines (Neurospec 2.0, Neurospec.org). The EEG data was segmented into epochs of 2 seconds with an overlap of 50% (1 second). After fast Fourier transformation the 95% spectral edge frequency (SEF95) for each infant was defined as the frequency between 0.5 and 30 Hz, below which 95% of the power was present. SEF95 was significantly affected by the EEG sampling frequency (high sampling frequency higher SEF95) [18]. Therefore the SEF95 was adjusted for this. Adjusted SEF95 within the 1^st^ quartile was considered an adverse outcome.

### Blood biomarkers

At the age of 6 and 64 hours (±1 h) 1 ml of blood was drawn from 123 infants with an indwelling arterial or venous line [18]. After inclusion of the last patient, the samples were shipped and analysed centrally (HaemoScan, Groningen, The Netherlands) without knowledge of the medical history of the infant. *S100beta* (50 μl) was assessed by ELISA: clone 1B2 monoclonal antibody (Abnova, Taipei, Taiwan) and biotinylated clone 8B10 (Hytest, Turku, Fi). Intra-assay variance is 4.6% and the lower level of quantification (LLOQ) is 39 pg/ml. *Brain fatty acid binding protein (BFABP) (*50 μl) was determined by ELISA with BFABP polyclonal capture antibodies and monoclonal detection antibody (HaemoScan). Intra-assay variance is 6.4% and the LLOQ is 150 pg/ml. *Neuroketal* (60 μl) was performed by competitive enzyme immunoassay (HaemoScan). Intra-assay variance is 10% and the LLOQ is 4.1 pg/ml. The laboratory analyses are described in detail elsewhere [18]. An increase in the biomarker concentration from 6 to 64 hours was considered as a marker of potential cerebral injury during the intervention period, therefore and adverse outcome was defined as an increase in the absolute value of the biomarker concentration from 6 hours to 64 hours within the 4^th^ quartile.

## Statistics

The median and inter quartile range of burden of cerebral hypo- and hyperoxia was determined. The infants were divided in groups according to a burden within or below the 4^th^ quartile of the burden of cerebral hypo- and hyperoxia, respectively. The infant characteristics were compared between the burden-groups using the chi-square test or independent t-test as appropriate. Odds ratios with 95% confidence intervals were determined for adverse outcomes for infants within the 4^th^ quartile of the burden of hypo- or hyperoxia versus infants in quartile 1–3. Thereafter univariate correlation analysis was conducted to determine the patient characteristics associated with the composite outcome of severe brain injury or death. Finally, a multiple logistic regression was used with the composite outcome as dependent variable, centre, gestational age above or below 26 weeks (the stratification variables used in the randomized SafeBoosC II trial), and intervention as forced entry independent variables, and the patient characteristics which had significant correlation to the composite outcome as independent variables in a backward stepwise elimination procedure (P-out 0.1).

The between twins inter-cluster correlation for the burden of hypo-and hyperoxia was low (0.02) [14]. Therefore, there was no need to exclude one infant from each twin-cluster thus data from all infants were included in the analysis.

None of the analyses reported here were specified in the SafeBoosC-II study protocol. The dichotomisation between one quartile and the three other quartiles was copied from previous work on biomarkers in our group [21], and chosen before any correlations were calculated.

The statistics was performed using IBM SPSS Statistics for Windows Version 20.0 (Armonk, New York, USA).

## Ethics

The SafeBoosC phase II trial was approved by each hospital’s research ethics committee (Hopital Femme Mere Enfants, Lyon, France; Rigshospitalet, Copenhagen, Denmark; La Paz University Hospital, Madrid, Spain; Cork University Maternity Hospital, Cork, Ireland; Wilhelmina Children’s Hospital, Utrecht, The Netherlands; Medical University of Graz, Graz, Austria; Fondazione IRCCS Ca’ Granda Ospedale Maggiore Policlinico, Milan, Italy; and Rosie Hospital, Cambridge University Hospitals, United Kingdom), and where required (Austria, Denmark, and France) by the competent authority responsible for medical devices. Written informed parental consent was mandatory before inclusion in the trial. The trial was conducted in compliance with the guidelines of the Declaration of Helsinki in its latest form and the International Conference on Harmonisation good clinical practice guidelines.

## Results

**Fig 1:**
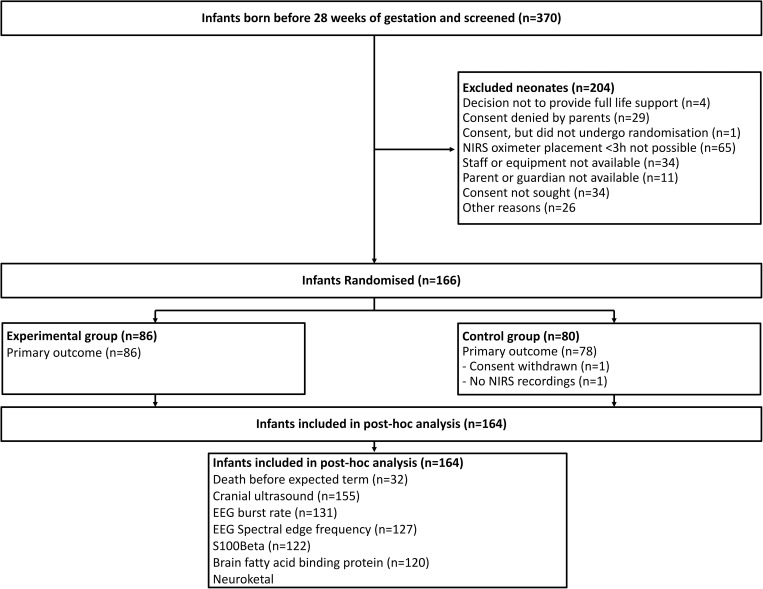
Flowchart. Flow of participants through the SafeBoosC II study.

One-hundred-and-sixty-six infants were included in the SafeBoosC II trial, Fig 1. Cerebral oximetry data was missing for two infants due to technical issues (n = 1) and withdrawal of consent (n = 1).

The median (min—max) burden of cerebral hypoxia was 30.6%hours (0.7–803.9). The limits for hypoxia quartile 4 were 99.3–803.9%hours and for quartile 1–3; 0.7–99.2%hours. The median (min—max) burden of cerebral hyperoxia was 1.2%hours (0.0–223.0). The limits for quartile 4 were 14.2–223.0%hours and for quartile 1–3; 0.0–14.1%hours. As expected according to the primary result of the SafeBoosC II trial [14], there were significantly fewer infants form the intervention group with a burden of hypoxia in the 4^th^ quartile, than in quartile 1–3, whereas the burden of cerebral hyperoxia within the 4^th^ quartile versus quartile 1–3, was unaffected by allocation in the SafeBoosC trial, Table 1. Male sex was associated with less cerebral hypoxia and more cerebral hyperoxia. Furthermore, gestational age was positively associated with cerebral hyperoxia whereas the other baseline characteristics were not associated with either cerebral hypo- or hyperoxia.

**Table 1:** Baseline characteristics and treatment during the first 72 hours of life according to burden of cerebral hypo- or hyperoxia split in the three lowest quartiles and the highest quartile.

	*Burden of hypoxia*			*Burden of hyperoxia*		
	Quartile 1 to 3	Quartile 4		Quartile 1 to 3	Quartile 4	
	n = 123	n = 41	P-value	n = 123	n = 41	P-value
**Baseline characteristics**						
Gestational age (week), mean (SD)	26.4 (1.2)	26.5 (1.5)	0.78	26.3 (1.3)	27.7 (1–0)	0.35
Gestational age below 26 weeks	38 (31)	14 (34)	0.7	42 (34)	10 (24)	0.025
Birth weight (gram), mean (SD)	847 (211)	875 (207)	0.47	849 (208)	872 (216)	0.54
Male sex	65 (53)	13 (32)	0.02	53 (43)	25 (61)	0.047
Twins	21 (17)	12 (29)	0.09	23 (19)	10 (24)	0.43
Antenatal steroids full course	82 (67)	31 (78)	0.2	88 (72)	25 (61)	0.18
Prolonged rupture of membranes	40 (33)	17 (36)	0.21	42 (34)	15 (38)	0.72
Maternal chorioamnionitis	6 (5)	5 (13)	0.1	9 (8)	2 (5)	0.59
APGAR-score <5 points at 5 minutes	21 (17)	8 (20)	0.69	20 (16)	9 (23)	0.38
Umbilical arterial pH, mean (SD)	7.32 (0.1)	7.31 (0.1)	0.62	7.32 (0.1)	7.29 (0.1)	0.13
SafeBoosC II intervention group	74 (60)	12 (29)	0.001	67 (54)	19 (46)	0.37
**Treatment during the first 72h of life**						
Surfactant treatment	90 (73)	35 (85)	0.11	90 (73)	35 (85)	0.11
Mechanical ventilation	79 (64)	30 (73)	0.29	80 (65)	29 (70)	0.5
Patent ductus arteriosus treatment	17 (14)	4 (10)	0.52	15 (12)	6 (15)	0.71
Use of vasopressors/inotropes	22 (18)	16 (40)	0.004	29 (24)	9 (22)	0.79
Any red blood cell transfusion	31 (26)	18 (45)	0.025	41 (35)	8 (20)	0.07
Corticosteroids	4 (3)	4 (10)	0.1	6 (5)	2 (5)	0.99

Values are numbers (percentages) unless stated otherwise. P-values have not been corrected for multiple comparisons.

**Fig 2:**
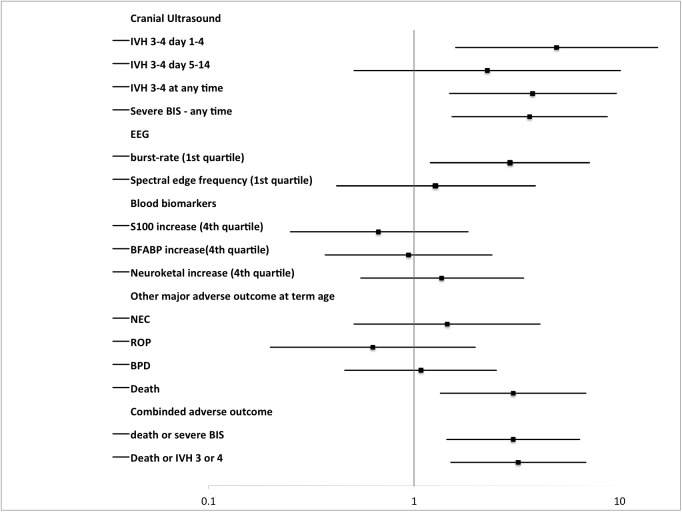
Risk for adverse outcomes for infants with a *burden of cerebral hypoxia* within or below the 4^th^ quartile. Odds ratio (OR) and 95% confidence interval.

**Fig 3:**
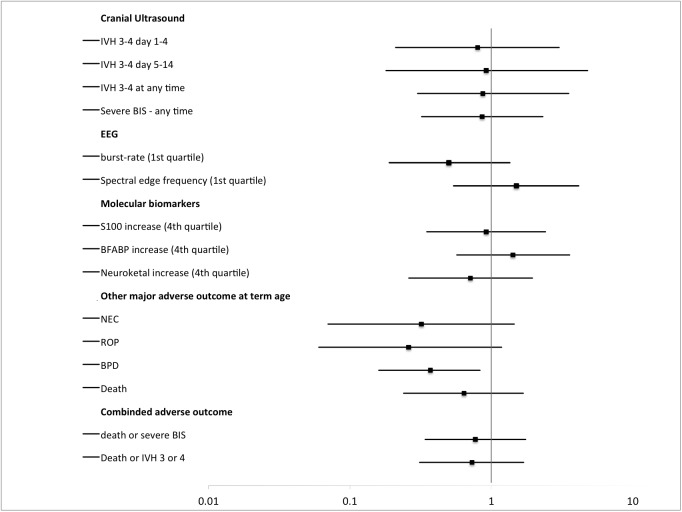
Risk for adverse outcomes for infants with a *burden of cerebral hyperoxia* within or below the 4^th^ quartile. Odds ratio (OR) and 95% confidence interval.

Serial ultrasound scans of 155 infants were available for evaluation by central reading. Twenty-seven (27/155) had severe brain injury, of which 22 had IVH grade III or PVHI. The remaining five infants had stroke (n = 2), cerebral atrophy (n = 2), or cerebellar haemorrhage (n = 1). Cerebral hypoxia was significantly associated with severe brain injury and death, Table 2. There was no significant association between cerebral hyperoxia and severe brain injury and death, Table 3. Cerebral hypoxia was significantly associated with early occurrence (day 1 to 4) of severe intracranial haemorrhages whereas there was no association between cerebral hypoxia and later occurrence (day 5 to day 14) of severe intracranial haemorrhages, Table 2. Cerebral hypoxia was also significantly associated with low EEG burst rates at 64 hours of age whereas there was no association between cerebral hypoxia and SEF95 at 64 hours of age. Of the 14 infants with early severe IVH ten (10) infants had EEG measurement and of these five (5) infants had burst rates within the 1^st^ quartile, for the infants without severe haemorrhages the number of recorded EEGs was 115 and hereof 26 had burst rates within the 1^st^ quartile (chi-square analysis between groups p = 0.12). The three plasma-biomarker-levels were not associated with cerebral hypoxia, Table 2. Cerebral hyperoxia was not associated with EEG burst rate, SEF95 or plasma biomarkers, Table 3. The odds ratios (ORs) and 95% confidence interval for adverse outcomes are illustrated in Figs 2 and 3.

**Table 2:** Distributions of early and late adverse outcomes of cranial ultrasound, EEG variables, blood biomarkers, term diagnoses, and death according to the *burden of cerebral hypoxia* within or below the 4^th^ quartile.

Burden of hypoxia	Quartile 1–3	Quartile 4				
	n = 123	n = 41	P-value	*P-value	OR	(95% CI)
Cranial ultrasound						
IVH 3–4 day 1–4	6/117	8/38	0.003	0.045	4.93	(1.59–15.30)
IVH 3–4 day 5–14	5/111	3/31	0.63	>0.95	2.27	(0.51–10.09)
IVH 3–4 at any time	11/117	11/39	0.003	0.045	3.77	(1.49–9.63)
Severe brain injury—any time	14/116	13/39	0.002	0.03	3.64	(1.53–8.69)
EEG variables time 64h						
Burst-rate in the 1^st^ quartile	21/103	12/28	0.015	0.23	2.93	(1.20–7.12)
Spectral edge frequency in the 1^st^ quartile	15/99	5/27	0.67	>0.95	1.27	(0.42–3.88)
Plasma biomarkers difference between 6 and 64h						
S100beta increase in 4^th^ quartile	25/92	6/30	0.43	>0.95	0.67	(0.25–1.83)
BFABP increase in 4^th^ quartile	24/89	8/31	0.90	>0.95	0.94	(0.37–2.39)
Neuroketal increase in 4^th^ quartile	22/92	9/30	0.51	>0.95	1.36	(0.55–3.41)
Other major adverse outcome at term age						
Necrotising enterocolitis	13/123	6/41	0.48	>0.95	1.45	(0.51–4.10)
Retinopathy of prematurity	18/123	4/41	0.43	>0.95	0.63	(0.20–1.98)
Bronchopulmonary dysplasia	52/104	14/27	0.86	>0.95	1.08	(0.46–2.51)
Death	18/123	14/41	0.006	0.09	3.03	(1.34–6.84)
Combined adverse outcome						
Death or severe brain injury	28/117	20/41	0.003	0.045	3.03	(1.44–6.38)
Death or IVH 3 or 4	25/118	19/41	0.002	0.03	3.21	(1.51–6.84)

Values are given as numbers of events / numbers of infants investigated for event.

* P-value after Bonferoni correction for multiple comparisons.

**Table 3:** Distributions of early and late adverse outcomes of cranial ultrasound, EEG variables, blood biomarkers, term diagnoses, and death according to the *burden of cerebral hyperoxia* within or below the 4^th^ quartile.

Burden of hyperoxia	Quartile 1–3	Quartile 4		
	n = 123	n = 41	P-value	*P-value	OR	(95% CI)
Cranial ultrasound						
IVH 3–4 day 1–4	11/116	3/39	0.74	>0.95	0.80	(0.21–3.01)
IVH 3–4 day 5–14	6/100	2/36	0.98	>0.95	0.92	(0.18–4.79)
IVH 3–4 at any time	17/117	5/39	0.79	>0.95	0.87	(0.30–3.52)
Severe brain injury—any time	21/117	6/38	0.76	>0.95	0.86	(0.32–2.31)
EEG variables time 64h						
Burst-rate in the 1^st^ quartile	27/95	3/36	0.17	>0.95	0.50	(0.19–1.35)
Spectral edge frequency in the 1^st^ quartile	13/91	7/35	0.43	>0.95	1.50	(0.54–4.14)
Plasma biomarkers difference between 6 and 64h						
S100beta increase in 4^th^ quartile	24/93	7/29	0.86	>0.95	0.92	(0.35–2.41)
BFABP increase in 4^th^ quartile	23/92	9/28	0.45	>0.95	1.42	(0.57–3.58)
Neuroketal increase in 4^th^ quartile	25/93	6/29	0.5	>0.95	0.71	(0.26–1.95)
Other major adverse outcome at term age						
Necrotising enterocolitis	17/123	2/41	0.12	>0.95	0.32	(0,07–1,45)
Retinopathy of prematurity	20/123	2/41	0.064	>0.95	0.26	(0.06–1.18)
Bronchopulmonary dysplasia	55/97	11/34	0.015	0.23	0.37	(0.16–0.83)
Death	26/123	6/41	0.36	>0.95	0.64	(0.24–1.68)
Combined adverse outcome						
Death or severe brain injury	38/120	10/38	0.53	>0.95	0.77	(0.34–1.75)
Death or IVH 3 or 4	35/120	9/39	0.46	>0.95	0.73	(0.31–1.69)

Values are given as numbers of events / numbers of infants investigated for event.

* P-value after Bonferoni correction for multiple comparisons.

Correlation analyses of the following variables showed a significant correlation (p<0.05) between gestational age, birth weight, clinical chorioamnionitis, surfactant, mechanical ventilation, use of vasopressors, blood transfusions, and the burden of cerebral hypoxia within the 4^th^ quartile on one hand and the composite outcome of severe brain injury or death on the other. These variables were included in the multiple logistic regression model with backward stepwise elimination. As described in the methods, centre, gestational age below 26 weeks, and intervention were forced-entry variables in the model. The following variables remained statistically significant: intervention (OR (95% CI) 0.29 (0.12–0.69; p = 0.003,)), gestational age below 26 weeks (3.33 (1.38–8.06; p = 0.007)), use of vasopressors (3.26 (1.26–8.44; p = 0.014)), and blood transfusion (2.97 (1.22–7.23; p = 0.016)). There was no centre-effect.

## Discussion

This post hoc analysis shows that early burden of cerebral hypoxia, but not hyperoxia, is associated with a reduction of brain electrical activity, severe brain injury (especially early IVH grade III and PVHI), and death. There were no significant associations between the burden of cerebral hypo- and hyperoxia and the three blood biomarkers. Multiple logistic regressions showed significant associations between intervention, gestational age below 26 weeks, use of vasopressors, and blood transfusion on one hand and the composite adverse outcome severe brain injury or death on the other.

Low cerebral oxygenation has previously been associated with IVH and lower developmental quotients at 2-year follow up [8–10]. In a study by Noori et al, involving 22 extremely preterm infants, lower levels of cerebral oxygenation, cardiac output, and cerebral hypoperfusion were found prior to the development of IVH II and PVHI [8]. In term piglet models, however, while low cerebral oxygenation when accompanied by low cerebral blood flow caused permanent brain damage, prolonged cerebral hypoxia alone seemed to be of less importance [22,23]. Piglets had to be exposed to cerebral hypoxia as low as 30–35% for several hours before significant histological damage appeared [23].

In the present analysis the burden of cerebral hypoxia was associated with decreased EEG burst rates. This is in agreement with a previous demonstration of an association between low cerebral blood flow and suppressed EEG in preterm infants [24]. EEG burst rate is decreased in infants with severe IVH [25,26] and suppressed EEG is correlated to adverse developmental outcome [27,28]. In the present analysis we did not find significantly more infants with low burst rates and early severe IVH grade III or PVHI.

The three blood biomarkers measured in the SafeBoosC II trial were not significantly associated with either cerebral hypo- or hyperoxia. We did not register whether the samples were drawn from arterial or venous line, therefore we are unable to determine whether the concentrations of the blood biomarkers differ between arterial and venous blood.

S100beta is a calcium binding protein present in high concentration in Schwann cells and astrocytes [29] and is released to the systemic circulation after cerebral damage. S100beta is an established marker of brain injury in adult trauma patients [30] and the levels of S100beta in blood and urine from term and preterm infants has been associated with the severity of both hypoxic ischemia and IVH [31–33]. And in a study including 64 term and late preterm infants the levels of S100beta was significantly negatively correlated to NIRS-values [34]. However a study by Rogers et al [35] including 130 extremely preterm infants reporting no associations between the S100beta levels and severe intracranial haemorrhages. S100Beta has also been investigated as a potential biomarker of brain injury in paediatric patients undergoing cardiac surgery–the infants with unfavourable neurological outcome 12 months after surgery had significantly lower cerebral NIRS values during surgery, whereas the levels of S100Beta did not differ [36]. We evaluated the effects of the accumulated burden of hypoxia during the first 72 hours of life rather than correlating specific NIRS-values to the levels of S100beta, and therefore we cannot determine whether the S100beta levels measured in the SafeBoosC II study were correlated to the NIRS-levels. We found no differences between the levels of S100beta between the 4th quartile of cerebral hypoxia where the proportion of infants with severe IVH was highest vesus the three lowest quartiles of cerebral hypoxia. The conflicting results of the associations between S100beta and severe intracranial haemorrhages make the clinical value of this biomarker questionable and further research is needed to evaluate whether S100beta is suitable as an early biomarker of brain injury in extremely preterm infants.

BFABP, a brain-specific marker, is rapidly released from astrocytes as a response to ischemia, mechanical and oxidative brain damage [37]. BFABP was chosen as a potential biomarker for brain injury in the SafeBoosC II trial, as it is known to rapidly increase in hypoxic-ischaemic stroke patients, and the high levels persist for several days after an event [37]. BFABP might be a more sensitive marker of minor traumatic brain injury than S100beta [38] and is elevated in patients with various neurodegenerative diseases[39]. A study involving 57 patients undergoing cardiac surgery reports that neither S100beta nor BFABP had any clear prognostic value for postoperative cognitive dysfunction [40]. We found no association between BFABP and the burden of cerebral hypo- or hyperoxia–this might be because of the different nature of the acute and severe local hypoxic-ischemia occurring in stroke patients compared to the global relative cerebral hypoxia measured in the SafeBoosC II trial. Our study ads information to the current litterateur of BFABP and brain injury–suggesting that there is no associations between the burden of cerebral hypoxia at 72h of age and the increase of the levels of BFABP from 6 to 64h of life. Further research on the associations between BFABP and brain injury in extremely preterm infants is needed.

Neuroketals are compounds produced by free radical induced peroxidation of docosahexenoic acid, have been associated with white matter damage on MRI in preterm infants [41] and are solely present in the brain [42]. Neuroketal is mainly released as a reaction to cerebral oxidative stress, and may due to their reactivity be involved in formation of protein cross-links, which is a common feature in neurodegenerative diseases, where neuroketal is known to be increased [43]. Neuroketal has been reported elevated in cerebrospinal fluid during the first 3 weeks of life infants with white matter damage on MRI performed at term equivalent age [41]. We expected that the levels of neuroketal would be increased in the infants with cerebral hyperoxia in the 4^th^ quartile, but that was not the case. This might be explained by the early measure of neuroketal– 6 to 64 hours of life and the fact that the level of cerebral hyperoxia in the SafeBoosC phase II trial was low [14]. In addition neuroketal is still only an experimental biomarker of cerebral injury and might not be a good marker of early acute brain injury in extremely preterm infants. Whether neuroketal in the future can serve as a marker of later white matter injury in extremely preterm infants remains to be determined.

### Limitations

The explorative post hoc analysis of the SafeBoosC II trial data presents the association between the early cerebral oxygenation and short-term adverse cerebral outcomes and death in extremely preterm infants. However, this study has some limitations. Most importantly, cranial ultrasound was conducted at pre-specified days of life, but the exact timing of IVH grade III and PVHI and other severe brain damages is not available and therefore we cannot know if cerebral hypoxia preceded IVH or vice versa. However, one small study in very preterm infants recently identified a significantly lower regional cerebral oxygenation during the early transition in infants who later developed IVH versus the infants who did not [44]. Similarly, EEG was only recorded once. While the timing at 64 hours of age was expected to assess the potential effects of the accumulated burden over the intervention period, finer details of preceding or concurrent cerebral hypoxia could not be extracted, such as the relative significance of longer periods of moderate hypoxia versus peaks of severe hypoxia. We did not collect data on continuous blood pressure, arterial oxygen saturation, or CBF, which may have contributed to both low cerebral NIRS values and severe brain damage [8,22,23].

The analyses are based on a dataset from our randomised clinical trial; therefore, the multiple logistic regression models were adjusted for the stratification variables (centre and gestational age below 26 weeks), as well as the randomisation indicator (experimental vs. control group). As the burden of hypoxia was reduced by 50% in the experimental group compared with the control group [14], it was therefore not surprising that adjusting for the randomisation indicator did reduce the statistical significance of the burden of hypoxia. We do not think that this changes the main conclusion of the study. On the other hand, the statistical significance of the randomisation indicator means that the risk of severe brain injury or death in the experimental group of the SafeBoosC-II trial was less in the experimental group than in the control group when adjusted for a number of other factors. However, neither the composite outcome nor the particular statistical analyses were specified in the trial protocol. This post-hoc finding must therefore be interpreted conservatively. Finally, the 2-year follow up of the infants included in the SafeBoosC II trial will further explore if cerebral hypoxia and/or the intervention is related to patient-relevant outcomes such as psychomotor deficit. Larger randomised clinical trials investigating possible patient-relevant benefits of continuous NIRS monitoring in extremely preterm infants is needed before the method is implemented as standard care in this population.

### Conclusions

Our analysis is currently the largest dataset published on cerebral oxygenation in extremely preterm infants providing data on short-term neurological outcomes. The results support the previous findings of associations between low cerebral oxygenation in extremely preterm infants during the first days of life and EEG suppression, severe intracranial haemorrhage, and death. However, our analyses are exploratory and we were unable to determine which came first: cerebral hypoxia or severe intracranial haemorrhage. We did not find any evidence that cerebral hyperoxia is associated with either death or severe brain damage. We found no associations between the burden of cerebral hypo- or hyperoxia and the three blood biomarkers (S100beta, BFABP, and Neuroketal).

## Supporting information

S1 Appendix The SafeBoosC Phase II protocol. (PDF)

S2 Appendix Consort 2010 Checklist. (DOC)
