# Supplementary material for: Early biomarkers of brain injury and cerebral hypo- and hyperoxia in the SafeBoosC II trial
Source: PLoS One. 2017 Mar 22;12(3):e0173440. doi: 10.1371/journal.pone.0173440 (PMC5362210; doi:10.1371/journal.pone.0173440)
Supplement: S1 Appendix — (PDF) [file pone.0173440.s001.pdf]

Clinical Investigation Plan

SafeBoosC

Safeguarding the brain of our smallest children

– an investigator-initiated randomised, blinded, multinational, phase II feasibility clinical trial on near-infrared spectroscopy monitoring combined with defined treatment guidelines versus standard monitoring and treatment as usual in premature infants

SafeBoosC phase II

**CIP number:**	SB010512
**Trial phase**	Phase II
**ClinicalTrials.gov:**	NCT01590316
**CTU number:**	SafeBoosC-CT-78
**CIP date (version):**	18.12.13 (Version 4.1)
**Sponsor:**	Region Hovedstaden<br>Department of Neonatology 5024, Rigshospitalet,<br>Blegdamsvej 9, 2100 Copenhagen Ø, Denmark<br>Phone: +45 3545 1326, Fax: +45 3545 5025<br>Email: Gorm.Greisen@regionh.dk
**Study site:**	Multicentre; international

Revision history

Version	Author	Date	Changes
1.0		29.082011	Initial Version,<br>Submitted to Danish Research Council
2.0	Gorm Greisen & Berit Grevstad	29.11.2011	No substantial changes<br>Submitted to ECRIN,
3.0	Simon Hyttel Sørensen & Gorm Greisen	16.03.2012	Adapted to Clinical Investigation Plan (CIP), Minor statistical adjustments according to ECRIN Scientific Board recommendations<br>Submitted to Competent Authorities and Ethics Committee
3.1	Simon Hyttel Sørensen	24.04.2012	Non-substantial changes: Inclusion criteria: Possibility to place cerebral NIRS oximeter within 3 hours after birth, according to CA directions, re-submitted to CA.
3.2	Gorm Greisen & Berit Grevstad	31.05.2012	Non-substantial changes: Urine samples only to be collected if catheter is already in place or can be done as part of routine non-invasive urine collection, FINAL signed, re-submitted to CA
3.3	Berit Grevstad	22.08.2012	Non-substantial changes: Decrease in blood volume requested collected.<br>Less restricted storing conditions for the blood and urine samples: -80ºC ±10 ºC.<br>Triplets cannot be included as no site has three concealing boxes available.
4.0	Gorm Greisen<br>Per Winkel<br>Anne Mette Plomgaard<br>Janus Engstrøm<br>Simon Hyttel Sørensen<br>Berit Grevstad	25.03.2013	Non-substantial changes:<br>• New member of the DMSC, replacing Cuno Uiterwaal due to potential conflict of interest, p. 9<br>• New German site replacing PI Claudia Roll, p. 7<br>• Re-positioning of the NIRS monitor, according to the unit practice.<br>• Appendix A: “Blood pressure low in normal range, consider:” has been changed to “Blood pressure below the normal range or even low in the normal range”<br>• MRI is to be conducted at 40-44 weeks of gestational age<br><br>Substantial changes:<br>• Now including both twins in the final analysis: Since both will be exposed to the potential risk of the trial it could potentially become an ethical issue if not including both twins in the analysis.<br>• Allowing enrolment of one twin of a set, if conditions do not allow enrolling both.<br>• Triplets cannot be included as no site has three concealing boxes available, but either one or two of the triplets can if equipment is available<br>• Targeted no. of patients adjusted from 150 to now 166 to compensate for the loss of degrees of freedom.

SafeBoosC phase II

			• The number of secondary outcomes is reduced and the statistical analysis plan adapted.<br>• Biomarker outcomes are the 64 hour values. A statistical analysis including the 64 hours and the 6 hours samples as well is not possible with twins. However, 6 hour samples must still be collected for exploratory analysis if at all possible.
4.1	Simon Hyttel Sørensen<br>Berit Grevstad	18.12.2013	Non-substantial change:<br>• P. 19: Correction of confidence interval

Summary

Background

25,000 infants are born extremely preterm every year in Europe. This group of infants carries a high risk of death and subsequent cerebral impairment for the infant, especially in the first 72 hours of life. Mortality is about 20%, and about 25% of survivors live with either cerebral palsy or low intelligence quotient. Preventative measures are keys to reducing mortality and morbidity in this population. There is evidence that the cerebral oxygenation time spent out of range (time with hypoxia or hyperoxia) is associated with poor outcome in infants. Near-infrared spectroscopy (NIRS) has been used to monitor tissue oxygenation since the mid-1980s, and quantification of oxygenation ($rStO_2$) in a percentage from 0 to 100% has been possible for 10 years. From almost 400 preterm infants normal ranges of $rStO_2$ has been determined to be from 55% to 85%. Still, there are no clinical trials and thus no solid evidence of the clinical utility of NIRS in preterm infants. Thus, research on the benefits and harms of cerebral monitoring using NIRS as a part of clinical management of premature infants is much needed.

Objectives

The primary objective of the SafeBoosC trial is to examine if it is possible to stabilise the cerebral oxygenation of extremely preterm infants during the first 72 hours of life through the application of cerebral NIRS oximetry and implementation of an $rStO_2$-specific clinical treatment guideline. We hypothesise that by using the specified treatment guideline to respond to cerebral monitoring readings outside the target range, we would reduce the burden of hypo- and hyperoxia and consequently reduce brain injury.

Trial design

This is an investigator-initiated randomised, blinded, multinational, phase II feasibility clinical trial involving preterm infants from 12 European countries.

Inclusion criteria

The inclusion criteria are: neonates born more than 12 weeks preterm (gestational age up to 27 weeks and 6 days); decision to conduct full life support; parental informed consent; and cerebral NIRS oximeter placed within 3 hours after birth.

Sample size

With a 50% reduction of the area outside the normal range of oxygenation in %hours in the experimental group compared to the control group as the minimal clinically significant difference, a standard deviation of the area outside the normal range of 83.2 %hours, a type I

SafeBoosC phase II

error (alpha) of 5%, and a type II error of 0.05 (power of 95%) inclusion of 83 preterm infants in the experimental group and 83 preterm infants in the control group is required..

Intervention

The premature infants will be randomised into one of two groups (experimental or control). Common is that both groups will have a cerebral oximeter monitoring device placed within three hours after birth. In the *experimental group*, the cerebral oxygenation reading is visible, and the infant will be treated accordingly using a defined treatment guideline. In the *control group*, the cerebral oxygenation reading is NOT visible, and the infant will be treated as usual.

Trial duration

Monitoring by cerebral oximeter will be started as soon as possible and within 3 hours after birth and the intervention will last for 72 hours. Thereafter, each neonate will be followed up at term date (approximately three months after birth) and at 24 months after term date.

Outcome measures

The primary outcome is the burden of hypo- and hyperoxia in %hours during the first 72 hours after birth. The secondary outcomes are brain activity on an amplitude-integrated electroencephalogram (aEEG),cerebral ultrasound score (cUS), and all cause mortality at term date (approximately three months after birth). The exploratory outcomes are blood biomarkers (brain fatty acid binding protein (BFABP), neuroketal, and S100β), serious and non-serious adverse reactions (SARs), burden of hypoxia, burden of hyperoxia, neonatal morbidities including bronchopulmonary dysplasia (BPD), necrotising enterocolitis (NEC), and retinopathy of prematurity (ROP), brain injury score on magnetic resonance imaging (MRI), number of therapies implemented during the intervention, physiological variables (mean blood pressure (BP), pulse oximeter oxygen saturation (SpO_2), and partial pressure of carbon dioxide (pCO_2)), and psychomotor impairment according to neurodevelopmental scales at 24 months after term date.

SafeBoosC phase II

Safety

Predefined serious adverse reactions, and suspected unexpected serious adverse reactions (SUSARs) will be recorded and reported to the appropriate competent authorities and ethics committees.

Ethical considerations

The approval from the relevant ethics committees will be sought. Parental informed consent will be obtained prior to randomisation. The trial will be conducted in compliance with the guidelines of the Declaration of Helsinki in its latest form and the International Conference on Harmonisation good clinical practice guidelines (ICH GCP). Procedures will be established to prevent and/or minimise risk of complication for participants, such as complications related to the device and the treatment guideline includes only interventions that are commonly used during intensive care in this population.

SafeBoosC trial flow chart

SafeBoosC-Phase II trial

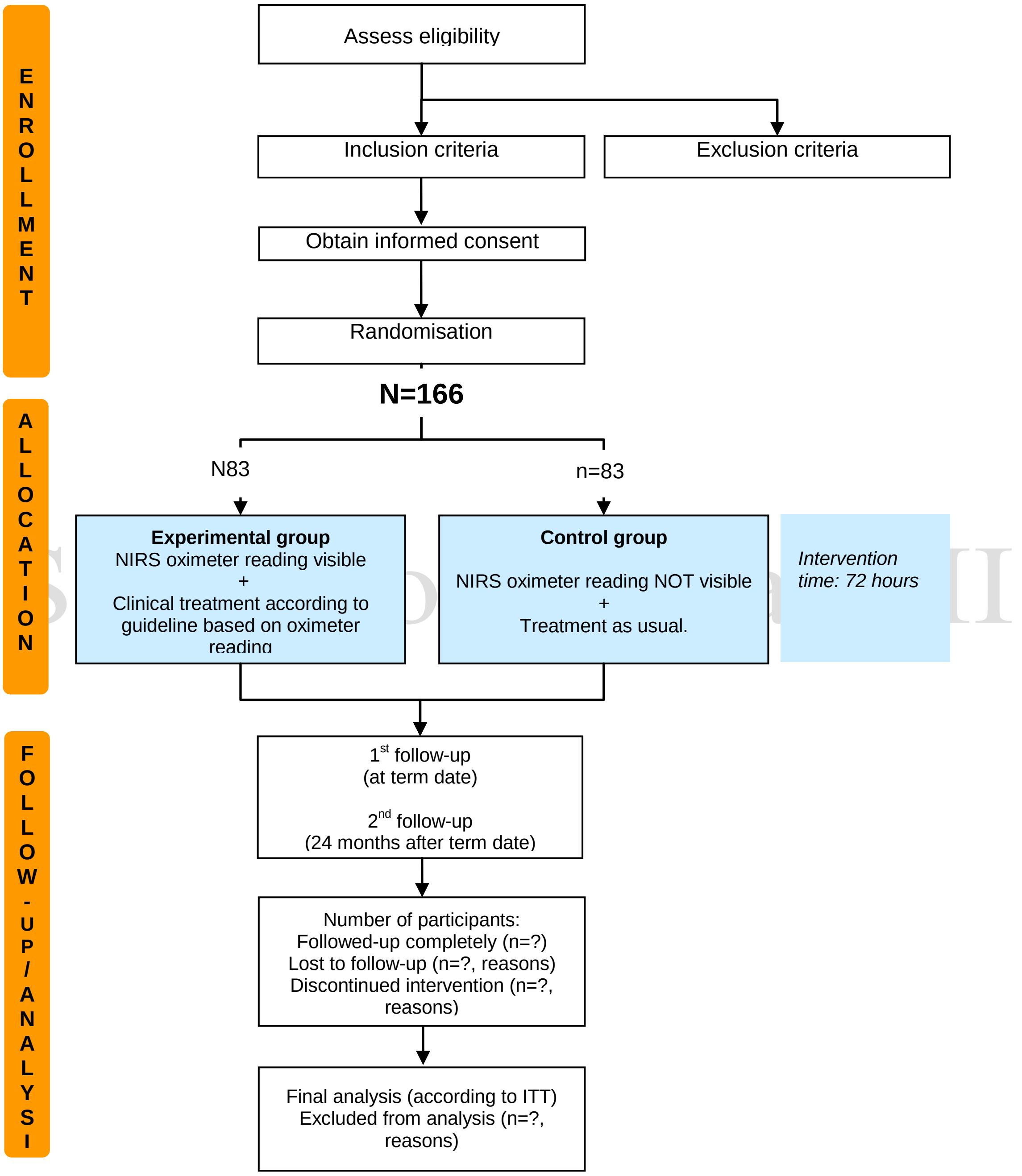

Source: adapted from the CONSORT Statement, 2010

Participating centres

Sponsor-investigator

Region Hovedstaden<br>Department of Neonatology 5024,<br>Rigshospitalet, Blegdamsvej 9, 2100<br>Copenhagen Ø, Denmark<br>Phone: +45 3545 1326, Fax: +45 3545 5025<br>Email: greisen@rh.dk<br>Gorm Greisen	DK

National coordinators

Medical University of Graz,<br>Gerhard Pichler	AT
Katholieke Universiteit Leuven,<br>Gunnar Naulaers	BE
University Hospital Zurich,<br>Cornelia Hagmann	CH
Universitätsklinikum Tübingen,<br>Axel Franz	DE
University of Copenhagen,<br>Gorm Greisen	DK
La Paz University Hospital (HULP),<br>Adelina Pellicer	ES
Hospices Civils de Lyon, Service de<br>Neonatologie, Femme-mere-enfant Hospital,<br>Olivier Claris	FR
University College Cork,<br>Gene Dempsey	IE
Università di Milano,<br>Monica Fumagalli	IT
Universitair Medisch Centrum Utrecht,<br>Frank van Bel	NL
Uppsala Universitet,<br>Lena Hellström-Westas	SE
Cambridge University Hospitals NHS<br>Foundation Trust,<br>Topun Austin	UK

SafeBoosC phase II

Steering Committee Members

Gerhard Pichler<br>Department of Pediatrics, Medical University of Graz<br>Auenbruggerplatz 30, Graz, Austria<br>Phone: +0316 38580520<br>E-mail: pichler.gerhard@klinikum-graz.at	AT	Olivier Claris<br>Hospices Civils de Lyon, Service de Neonatologie, Femme-mere-enfant Hospital, Department of Neonatology<br>Hospital Femme Mere Enfants, 59 Boulvard Pinel 69500 Bron France<br>Phone: +33 (0) 427855283<br>E-mail: olivier.claris@chu-lyon.fr	FR
Gunnar Naulaers<br>Katholieke Universiteit Leuven<br>Herestraat 49, 3000 Leuven, Belgium<br>Phone: tel. +3216343213 or +32 16 343211, fax +32 16 343209<br>E-mail: gunnar.naulaers@uz.kuleuven.ac.be	BE	Gene Dempsey<br>University College Cork<br>University College Cork, College Road, Cork, Ireland<br>Phone: +353 21 492 0525<br>E-mail: g.dempsey@ucc.ie	IE
Cornelia Hagmann<br>Clinic of Neonatology<br>University of Zurich<br>8091 Zurich, Switzerland<br>Phone: +41 44 255 53 98<br>E-mail: cornelia.hagmann@usz.ch	CH	Monica Fumagalli<br>Department of Maternal and Pediatric Sciences, Università di Milano<br>Fondazione IRCCS Ca' Granda Ospedale Maggiore Policlinico Milan – Università degli Studi di Milano, Via della Commenda 12, IT-20122 Milan, Italy<br>Phone: +39 02 55 032 951<br>E-mail: monica.fumagalli@mangiagalli.it	IT
Martin Wolf<br>Biomedical Optics Research Laboratory<br>Clinic of Neonatology, University Hospital Zurich<br>Frauenklinikstr 10, 8091 Zurich, Switzerland<br>Phone: +41 44 255 5346, Fax: +41 44 255 4442<br>E-mail: martin.wolf@usz.ch	CH	Frank van Bel<br>Universitair Medisch Centrum Utrecht<br>Wilhelmina Children's Hospital<br>KE 04.123.1<br>PO Box 85090,<br>3508 AB Utrecht, The Netherlands<br>Phone: +31-88-7554545<br>E-mail: F.vanBel@umcutrecht.nl	NL
Axel Franz<br>Kinderarzt, Neonatologe<br>Kinderkardiologe, Pädiatrische Intensivmedizin<br>Abt. Kinderheilkunde IV, Neonatologie<br>Universitätsklinikum Tübingen<br>Calwerstr. 7<br>72076 Tübingen, Germany<br>phone: +49 7071 29 82211<br>fax: +49 7071 29 5923<br>E-mail: Axel.Franz@med.uni-tuebingen.de	DE	Wim van Oeveren<br>Haemoscan B.V.<br>Stavangerweg 23<br>Groningen<br>9723 JC<br>The Netherlands<br>Phone : +31(0)646181604<br>Fax nr +31 (0)847269747<br>E-mail: mail@haemoscan.com	NL
Gorm Greisen<br>Department of Neonatology 5024, Rigshospitalet, Blegdamsvej 9, 2100 Copenhagen Ø, Denmark<br>Phone: +45 3545 1326,<br>E-mail: greisen@rh.dk	DK	Lena Hellström-Westas<br>Dept of Women's and Children's Health<br>Uppsala Universitet,Dept of Neonatology, University Hospital, 751 85 Uppsala, Sweden<br>Phone: +46 18 6114877, mobile: +46 73 3916330<br>E-mail: lena.westas@kbh.uu.se	SE
Christian Gluud<br>Copenhagen Trial Unit<br>Centre for Clinical Intervention Research<br>Department 3344, Rigshospitalet<br>Blegdamsvej 9, DK-2100 Copenhagen<br>Phone: +45 3545 7171, Fax: +45 3545 7101<br>E-mail: cgluud@ctu.dk	DK	Topun Austin<br>Rosie Maternity Hospital, Cambridge University Hospitals NHS Foundation Trust<br>Hills Road<br>Cambridge, CB2 0SW, United kingdom<br>Phone:<br>E-mail: topun.austin@addenbrookes.nhs.uk	UK

SafeBoosC phase II

Adelina Pellicer<br>La Paz University Hospital (HULP)<br>Department of Neonatology, La Paz University Hospital, Paseo de la Castellana 261, 28046 Madrid, Spain<br>Phone +34 917277416, Fax: +34 917277362<br>e-mail: apellicer.hulp@salud.madrid.org	ES		

SafeBoosC phase II

Executive Committee Members

Martin Wolf<br>Biomedical Optics Research Laboratory<br>Clinic of Neonatology<br>University of Zurich<br>8091 Zurich, Switzerland<br>Phone: +41 44 255 5346, Fax: +41 44 255 4442<br>E-mail: martin.wolf@usz.ch	CH	Adelina Pellicer<br>La Paz University hospital (HULP)<br>Department of Neonatology, La Paz University Hospital, Paseo de la Castellana 261, 28046 Madrid, Spain<br>Phone +34 917277416, Fax: +34 917277362<br>e-mail: apellicer.hulp@salud.madrid.org	ES
Gorm Greisen<br>Department of Neonatology 5024, Rigshospitalet, Blegdamsvej 9, 2100 Copenhagen Ø, Denmark<br>Phone: +45 3545 1326,<br>Email: greisen@rh.dk	DK	Frank van Bel<br>Universitair Medisch Centrum Utrecht<br>Wilhelmina Children's hospital<br>KE 04.123.1<br>PO Box 85090,<br>3508 AB Utrecht, The Netherlands<br>Phone: +31-88-7554545<br>E-mail: F.vanBel@umcutrecht.nl	NL
Christian Gluud<br>Copenhagen Trial Unit<br>Centre for Clinical Intervention Research<br>Department 3344, Rigshospitalet<br>Blegdamsvej 9, DK-2100 Copenhagen, Denmark<br>Phone: +45 3545 7171, Fax: +45 3545 7101<br>E-mail: cgluud@ctu.dk	DK	Lena Hellström-Westas<br>Dept of Women's and Children's Health<br>Uppsala Universitet<br>Dept of Neonatology, University Hospital, SE-751 85 Uppsala, Sweden<br>Phone: +46 18 6114877, mobile:<br>+46 73 3916330<br>E-mail: lena.westas@kbh.uu.se	SE

SafeBoosC phase II

Data Monitoring and Safety Committee

Jan Miletin<br>Department of Paediatrics and Newborn Medicine, Coombe Women and Infants University Hospital, Dublin, Ireland.<br>Phone: +353-(0)87 9819668<br>E-mail: miletinj@yahoo.com	IE
Armin Koch<br>Institut für Biometrie<br>Medizinische Hochschule Hannover<br>Carl-Neuberg-Str. 1<br>30625 Hannover<br>Germany<br>Phone: +49 511.532-4419<br>E-mail: Koch.Armin@mh-hannover.de	NL
Heike Rabe (Chair)<br>Trevor Mann Baby Unit, Brighton and Sussex University Hospitals NHS Trust, United Kingdom<br>Phone +44-(0)1273-696955 ext 4195/4296, fax +44-(0)1273-664435<br>E-mail Heike.Rabe@bsuh.nhs.uk	UK

Clinical laboratories and other collaborators

Manon Benders and Cornelia Hagmann<br>MRI and CUS<br>Department of Neonatology,<br>KE 04.123.1<br>Universitair Medisch Centrum Utrecht<br>Wilhelmina Children's Hospital<br>KE 04.123.1<br>PO Box 85090,<br>3508 AB Utrecht, The Netherlands<br>Phone: +31-88-7554545<br>E-mail: m.benders@umcutrecht.nl	NL	Wim van Oeveren<br>Haemoscan B.V.<br>Stavangerweg 23-23<br>Groningen<br>9723 JC<br>The Netherlands<br>Phone : +31(0)646181604<br>Fax nr +31 (0)847269747<br>E-mail: mail@haemoscan.com	NL
Martin Wolf<br>Oximetry<br>Biomedical Optics Research Laboratory<br>Clinic of Neonatology, University Hospital Zurich<br>Frauenklinikstr 10, 8091 Zurich, Switzerland<br>Phone: ++41 44 2555346, Fax: ++41 44 2554442<br>Email: martin.wolf@usz.ch	CH	Lena Westas<br>aEEG<br>Dept of Women's and Children's Health, Uppsala Universitet<br>Dept of Neonatology, University Hospital, SE-751 85 Uppsala, Sweden<br>Phone +46-18-611 00 00 or 611 48 77<br>Phone: +46 18 6114877, mobile:<br>+46 73 3916330<br>E-mail: lena.westas@kbh.uu.se	SE
Per Winkel<br>Biostatistician<br>Copenhagen Trial Unit<br>Centre for Clinical Intervention Research<br>Department 3344, Rigshospitalet<br>Blegdamsvej 9, DK-2100 Copenhagen, Denmark<br>Phone: +45 3545 7171, Fax: +45 3545 7101<br>E-mail: pwinkel@ctu.dk	DK	Christian Gluud<br>Data Management<br>Copenhagen Trial Unit<br>Centre for Clinical Intervention Research<br>Department 3344, Rigshospitalet<br>Blegdamsvej 9, DK-2100<br>Copenhagen,Denmark<br>Phone: +45 3545 7171, Fax: +45 3545 7101<br>E-mail: cgluud@ctu.dk	DK

SafeBoosC phase II

Trial monitors

The GCP-unit at the Copenhagen University Hospital<br>Bispebjerg Hospital, Building 51, 3.sal<br>Bispebjerg Bakke 23, DK-2400 Copenhagen NV<br>Lotte Laursen<br>Tel: +45 3531 3890<br>E-mail: lotte.laursen@regionh.dk, http://gcp-enhed.dk/kbh	DK	Instituto di Recerche Farmacologiche Mario Negri<br>Department of Cardiovascular Research<br>Via La Masa 19, 20156 Milano, Italy<br>Lucia Musicco<br>lucia.musicco@marionegri.it	IT
TBA	NL	Udo Eggenreich<br>CIS-Services - Eggenreich & Gschanes GmbH<br>Firmenbuch: Landesgericht für ZRS Graz; FN 338963-a<br><br>Grazbachgasse 53/7/49<br>8010 Graz, Austria<br><br>Fon +43 316 890 430<br>Fax +43 316 890 430 15<br>Mobile +43 699 1292 3230<br>Email u.eggenreich@cis-services.co.at	AT

TBA	BE	Sophie Lewis<br>sophie.lewis@addenbrookes.nhs.uk	UK
María Yllescas<br>Coordinadora UCICEC - IdiPAZ<br>Hospital La Paz<br>Paseo de La Castellana, 261<br>Edificio de Maternidad - 2ª planta<br>28046 - Madrid<br>Telf: +34 91 727 75 58<br>Fax: +34 91 207 18 76<br>Email: maria.yllescas@idipaz.es	ES	Center for Paediatric Clinical Studies<br>http://www.medizin.uni-tuebingen.de/kinder/en/research/cpcs/	DE
TBA	CH	Laurie Adellach<br>laurie.adellach@chu-lyon.fr	FR
TBA	SE	Jackie O Leary,<br>Neonatal Brain Research Group, UCC.	IE

SafeBoosC phase II

Table of contents

Summary ... 3

SafeBoosC trial flow chart ... 5

Participating centres ... 6

Steering Committee Members ... 7

Executive Committee Members ... 9

Data Monitoring and Safety Committee ... 9

Clinical laboratories and other collaborators ... 10

Trial monitors ... 10

Table of contents ... 12

1. Introduction and background ... 17
1.1 The population and condition ... 17
1.2 Pathophysiology ... 17
1.2.1 The transition from foetal to infant circulation ... 17
1.2.2 Cerebral autoregulation ... 17
1.2.3 The vulnerable brain ... 18
1.2.4 Mechanisms of brain damage in preterm infants ... 18
1.3 Current clinical management ... 18
1.4 Assessment of brain injury and neurodevelopmental deficit ... 19
1.4.1 Ultrasound ... 19
1.4.2 aEEG ... 19
1.4.3 MRI ... 20
1.4.4 Biomarkers ... 20
1.4.5 Neurological developmental assessment tool ... 21
1.5 Cerebral oximetry monitoring ... 21
1.5.1 NIRS devices ... 22
1.6 Regional oxygenation saturation in preterm infants ... 23
1.7 Previous trials on similar research question ... 23
1.8 Trial rationale ... 24
1.9 Justification for the design of the clinical investigation ... 24
1.9.1 Rationale for use of INVOS Adult SomaSensors® as reference standard for mean values in neonates in the study ... 24
1.9.2 Rationale for the evaluation of the investigational devices ... 24

2. Trial objective and hypothesis ... 25

3. Trial design ... 25
3.1 Randomisation ... 26
3.2 Trial interventions ... 26
3.3 Duration ... 26
3.4 Blinding ... 26

4. Participants ... 27
4.1 Inclusion criteria ... 27
4.2 Exclusion criteria ... 27
4.3 Exclusion Period ... 27
4.4 Participant discontinuation and withdrawal ... 27
4.5 Recruitment feasibility ... 28

5. Interventions ... 28
5.1 Common to both groups ... 28

SafeBoosC phase II

5.2 Experimental group ... 28
5.3 Control group ... 28
5.4 Concomitant medication/treatment ... 29
5.5 Medical care after end of clinical investigation ... 29

6. Outcome measures ... 29
6.1 Primary ... 29
6.2 Secondary ... 29
6.3 Exploratory ... 29
6.4 Outcome assessment tools ... 30
6.5 Outcome assessment committee ... 30
6.6 Compliance with the CIP ... 30

7. Data collection and trial assessment schedule ... 30
7.1 Collection of trial data ... 30
7.1.1 NIRS device ... 30
7.1.2 Case record form ... 30
7.2 Trial assessment schedule ... 31

7.3 Research biobank ... 32

8. Assessment of safety ... 34
8.1 Adverse events and reactions ... 34
8.1.1 Definitions ... 34
8.1.2 Classification of causality ... 36
8.1.3 Recording and reporting of adverse events and reactions and device deficiencies ... 36
8.1.4 Justification for recording and reporting periodicity and modality ... 38
8.1.5 Timelines for recording and reporting ... 39
8.2 Cerebral NIRS monitoring device ... 39
8.3 Data Monitoring and Safety Committee ... 40
8.4 Suspension or premature termination of the clinical investigation ... 40

9. Ethical Considerations ... 41
9.1 Informed consent procedure ... 41
9.2 Risk of complication for participants ... 42
9.3 Benefit for participants ... 42

10. Statistical plan and data analysis ... 43
10.1 Sample size estimation ... 43
Figure 4: The distribution of the primary outcome burden of hypo-and hyperoxia expressed as %hours and transformed logarithmically ... 44
10.2 Data analysis and statistical methods ... 45

11. Data management ... 47
11.1 Data handling and archiving ... 47
11.2 Medical coding ... 48

12. Quality assurance ... 48
12.1 Monitoring ... 48
12.2 Device quality control ... 48

13. Trial and funding timeframe ... 48

14. Legal aspects ... 49
14.1 Finance ... 49
14.2 Participant insurance ... 49
14.3 Publication plan ... 49
14.4 Statements of compliance ... 49

15. Appendices ... 51

SafeBoosC phase II

15.1 Appendix A: Treatment guidelines and justifications 51
15.2 Appendix B: Procedure for assessment of chemical biomarkers 54
15.3 Appendix C: Procedures for assessment of aEEG/EEG 56
15.4 Appendix D: Procedures for MRI examination 57
15.5 Appendix E: Procedure for assessment of cranial ultrasound 60
15.6 Appendix F: Parental Information 62
15.7 Appendix G: Informed consent – the SafeBoosC phase II trial 62
15.8 Appendix H: Parental information - the SafeBoosC trial -MRI 62
15.9 Appendix I: Informed consent- the SafeBoosC trial -MRI 62
15.10 Appendix J: SafeBoosC - Data flow 62

16. References 64

SafeBoosC phase II

List of abbreviations

AE	Adverse events
aEEG	Amplitude-integrated electroencephalogram
AR	Adverse reaction
ASQ	Ages & Stages Questionnaires
AUC	Area under the curve
BFABP	Brain fatty acid binding protein
BP	Blood pressure
BPD	Broncopulmonary dysplasia
BSID-III	Bayley’s scale of infant development, third version
cPVL	Cystic periventricular leucomalacia
CIP	Clinical Investigation Plan
CRF	Case record form
CTU	Copenhagen Trial Unit
cUS	Cerebral ultrasound
Da	Dalton
EAR	Expected adverse reaction
eCRF	electronic case record form
EEG	Electroencephalogram
EDTA	Ethylenediaminetetraacetic acid
ELISA	Enzymelinked immunosorbent assay
ESAR	Expected serious adverse reaction
GA	Gestational age
GCP	Good clinical practice
H	Hydrogen
Hb	Haemoglobin
Hz	Hertz
IB	Investigator's Brochure
IBI	Interburst Interval
ICH	International Conference on Harmonization
ITT	Intention-to-treat
IVH	Intraventricular haemorrhage

SafeBoosC phase II

LLT	Lowest level term
MAR	Missing at random
MedDRA	Medical Dictionary for Regulatory Activities – a medical coding system
MI	Multiple imputation
MMRM	Mixed model with repeated measures
MNAR	Missing not at random
MRI	Magnetic resonance imaging
NEC	Necrotizing enterocolitis
NICU	Neonatal intensive care unit
NIRS	Near-infrared spectroscopy
O2	Oxygen
pCO2	Partial pressure of carbon dioxide
PDA	Patent ductus arteriosus
PPV	Positive predictive value
PT	Preferred term
PV-IVH	Periventricular-intraventricular haemorrhage
PVL	Periventricular leucomalacia
RCT	Randomised clinical trial
ROP	Retinopathy of prematurity
rStO2	Regional tissue oxygen saturation
S100β	Acidic calcium binding protein found in the nervous system
SAR	Serious adverse reactions
SAE	Serious adverse event
SafeBoosC	Safeguarding the brain of our smallest children
SD	Standard deviation
SOC	System organ class
SpO2	Pulse oximeter oxygen saturation
SUSAR	Suspected unexpected serious adverse reaction
Term date	Is defined as gestational age of 40 weeks. The term date will be approximately three months after birth for this extremely preterm population.
UAR	Unexpected adverse reaction
3D-MRI	Three-dimensional magnetic resonance imaging

SafeBoosC phase II

1. Introduction and background

1.1 The population and condition

Infants born more than 12 weeks preterm (extremely preterm) carry a high risk of death or long-term cerebral impairment. Currently, mortality is about 20%, and about 25% live with either cerebral palsy or low intelligence quotient (1). Every year 25,000 extremely preterm infants are born in Europe. Psychomotor impairment is the major cause of reduced quality of life and increased costs of medical care, rehabilitation, and special education in this population. Because of the long life expectancy of children, this is an important problem.

Unfortunately, prevention of preterm birth and its consequences has not been successful; the rate of extremely preterm birth is stable or even increasing. Although there are risk factors, such as multiple pregnancy, and previous preterm birth, most extremely preterm births occur in otherwise normal and healthy women.

1.2 Pathophysiology

1.2.1 The transition from foetal to infant circulation

The transition from foetal to neonatal life is a particular problem in the extremely preterm infant. In foetal life, blood circulation includes only minimal perfusion of the lungs due to a large right-to-left shunt through the foramen ovale to the left side of the heart and through the arterial ductus from the pulmonary artery to the descending aorta. At birth, increased oxygenation of the body results in a systemic vasoconstriction and increasing arterial blood pressure. This may become a problem since the immature myocardium is intolerant to increased afterload. Also, as the lung function improves, the resistance of the pulmonary vessels drops causing left-to-right shunting over the arterial duct, which increases the need for left ventricular output. The immature myocardium's ability to increase stroke volume is limited due to poor diastolic function. The increased afterload and possible left-to-right shunting may cause low systemic blood flow (2)

SafeBoosC phase II

1.2.2 Cerebral autoregulation

Autoregulation is the ability to keep the organ blood flow constant despite fluctuations in perfusion pressure. It is accomplished by regulation of the arterial tone so that low perfusion pressure results in vasodilation and high perfusion results in vasoconstriction. On the systemic level, organs such as the brain, heart, and adrenals are vital and autoregulation maintains normal organ blood flow when systemic blood flow is low, while non-vital organs (e.g., skin and kidney) vasoconstricts to direct the circulating blood to the vital organs.

Cerebral autoregulation has limited capacity and is thought to be particularly fragile in the immature brain (1). Pressure passive flow is a state where the blood flow follows the blood pressure. It is hypothesized that the potential large fluctuations in flow that this entails is a cause of cerebral haemorrhages in premature infant due to rupture of the immature blood vessels. It is a problem that, at the current state, it is not possible to identify the individual threshold systemic blood pressure below which cerebral blood flow begins to fall (3).

1.2.3 The vulnerable brain

All the organs are immature when an infant is born more than 12 weeks before term. The immaturity and functional limitations of the lungs, heart, intestine, kidneys, liver, and endocrine system all contributes to the acute problems of extremely preterm birth. The brain is special, however, in the sense that brain damage results in death or in neuropsychological deficits such as cerebral palsy, cognitive deficit, attention deficit disorder, and major psychiatric disorder. These damages result in long-term consequences for children after extremely preterm birth.

The most easily identifiable type of brain damage in extremely preterm birth is periventricular-intraventricular brain haemorrhage (PV-IVH). Its severity varies: in the mildest form, the haemorrhage is limited to the subependymal germinal matrix – possibly with a small intraventricular clot. The most severe form is a large periventricular haemorrhagic infarction primarily located in the central white matter in one or both hemispheres. This predicts a high probability of death or cerebral palsy and may result in hydrocephalus (4). Hydrocephalus needing surgical treatment carries a poor neurodevelopmental prognosis. Periventricular leucomalacia (PVL) is a non-haemorrhagic white matter damage. In the mildest form, the condition is non-cystic and predicts poor psychomotor development. The most severe form of PVL is when the condition becomes cystic (cPVL) 2-5 weeks after the damage is induced and is a strong predictor of cerebral palsy (5).

1.2.4 Mechanisms of brain damage in preterm infants

SafeBoosC phase II

The mechanisms of the brain damage in preterm infants are complex. Some of the mechanisms are evoked before birth or even before the start of delivery such as a foetal inflammatory response induced by infection ascending to the foetal membranes. Also, late effects such as insufficient nutrition and poor growth during the first months of life may play a role.

The days after birth, however, are likely to be of particular importance. This is the period of change from a state of low oxygen pressure ('Mount Everest in-utero') to a state of 'normoxaemia'. Moreover the circulatory adaption to birth is as described problematic in the preterm infant. Thus fluctuations in systemic blood flow are common during the first days of life.

The following postnatal factors have been shown or are thought to be associated with brain injury: respiratory distress syndrome (6), hypocapnia due to inadvertent hyperventilation (7), low blood pressure (8), perturbations in arterial and venous pressure (9), and also low cerebral blood flow (10). In addition, clinical and experimental evidence is suggesting that hyperoxygenation is dangerous due to lack of a developed antioxidant defence system (11).

An important common mechanism for these associations is disturbance of cerebral blood flow partly due to impaired cerebral autoregulation.

1.3 Current clinical management

Current standard of care of the extremely premature infants during their first 72 hours involves a number of different parallel interventions:

- *Respiratory support:* continuous positive airway pressure or mechanical ventilation is almost universal and surfactant is usually administered within the first 24 hours.
- *Haemodynamic support:* Before diagnosing a patent ductus arteriosus (PDA), prophylactic use of indomethacin can be used. Either indomethacin or ibuprofen can be used for the closure of a PDA. Fluid boluses, inotropics, or vasopressors are used to treat hypotension, although the level or targeted blood pressure is controversial (12).
- *Fluid balance/nutrition:* Close observation of hourly and daily estimations of in- and output, scheduled fluid administration, and blood sugar monitoring. Most infants initially receive full parenteral nutrition and will slowly be introduced to breast milk.
- *Monitoring*: Invasive/non-invasive blood pressure monitoring, continuous pulse oximetry, transcutaneous partial pressure of carbon dioxide (pCO2), and electrocardiographic monitoring with frequent measurements of arterial blood gases, electrolytes and temperature.

Treatment of the extremely premature infants has certainly improved over the last three decades despite great areas of unknown territory. However, the treatment of hypotension, the optimal arterial oxygen content, the optimal pCO2 level, and many other possible interventions are dealt with on more or less loose grounds. And while still more comprehensive monitoring is implemented in the intensive care of premature infants, an end-organ monitoring with sufficient high time resolution to guide evidence-based treatment interventions is lacking. Near infrared spectroscopy has the potential to become that monitor of the brain.

SafeBoosC phase II

1.4 Assessment of brain injury and neurodevelopmental deficit

1.4.1 Ultrasound

Cerebral ultrasound (cUS) is a standard tool for diagnosing conditions such as haemorrhage and hypoxic-ischaemic lesions. Furthermore, signs of brain atrophy at term equivalent age are associated with neurodevelopmental outcome in preterm infants (13). The pooled probability for a normal neuromotor outcome of a normal ultrasound was 94% (95% confidence interval (CI) 92% to 96%) and 82% (95% CI 79% to 85%) for a normal cognitive outcome. Additionally, for IVH Grade I-II, the probability of abnormal outcome was 9%, and for IVH Grade III 26% (95% CI 13% to 45%) (14). Parenchymal haemorrhagic infarction predicted an abnormal neurodevelopmental outcome with an increased risk positive predictive value (PPV) of 47% (95% CI 31% to 64%) (15). Cystic PVL was predictive of cerebral palsy with a PPV of 77% (95% CI 59% to 89%) (15). Cerebellar haemorrhage predicted abnormal outcome with a PPV of 71% (95% CI 42% to 90%) (16).

1.4.2 aEEG

Amplitude-integrated electroencephalography (aEEG) is a technique where the EEG signal from bilaterally placed electrodes is recorded and amplified. The signal is then passed through an asymmetrical band pass filter, which minimises noise and artefacts before it is displayed bedside. The aEEG is widely used in term infants with asphyxia. It has shown good predictive value (17) and is suitable for detection of seizure activity (18), cerebral haemorrhages (19) as well as the effects of variation in pCO2 and blood sugar in the first day of life in extremely preterm infants (20).

1.4.3 MRI

Three-dimensional magnetic resonance imaging (3D-MRI) of the newborn brain is helpful in identifying extra information about brain injury and maturation, which cannot always be visualised on cranial ultrasound. Furthermore, when using advanced post-processing techniques, it is possible to measure the volume of the total brain volume and of various brain structures, which might be helpful in identifying non-visual brain injury or differences. Differences in volume of different cerebral tissue classes and cortical folding and other brain maturation measurements can be precisely measured in a quantitative way to assess cerebral growth and maturation as well as in relation to sulci formation that has been described to be related to cognitive outcome. Thus, 3D-MRI provides us valuable information about brain growth/development and neurodevelopmental outcome.

1.4.4 Biomarkers

Earlier research demonstrates that several neuro-biomarkers are released into body fluids in response to perinatal asphyxia or hypoxic-ischaemic brain injury. Some of them have also been correlated to severity of hypoxic ischaemia and long-term neurological outcome in neonates within the first day of life. Moreover, the course of these biological markers could correlate with the process of hypoxic ischaemic injury and thus have a diagnostic value for brain injury in preterm neonates with hypoxic ischaemic injury. Serum can be analysed for the rise in the following chemical biomarkers: brain fatty acid binding protein (BFABP), neuroketal, and S100β.

SafeBoosC phase II

- *Brain fatty acid binding protein (BFABP)* is a 15 kDa protein, which is specific for brain tissue. It is released from asterocytes after mechanical damage, ischaemia and oxidative brain damage. BFABP is determined by means of enzyme-linked immunosorbent assay (ELISA) with BFABP specific monoclonal capture antibodies and polyclonal detection antibody in plasma/serum as well as urine. Due to its low concentrations, 100 µl is needed for a BFABP ELISA.

- *Neuroketal* is formed in the brain via the neuroprostane pathway during oxidation of docohexaenoic acid. Neuroketals rapidly adduct to lysine, and these crosslinks induce neurodegenerative disease. Neuroketal thus contributes to the injurious effects of oxidative pathologies in the brain. Determination of neuroketals is performed by competitive enzyme immunoassay in 100 µl of plasma, serum or urine.

- *S100β* is a marker for brain damage. S100 β is an acidic calcium-binding protein found in the nervous system of vertebrates. It is a dimer of α and β subunits. As such, S100β, which consists of two β subunits, is present in high concentration in glial and Schwann cells. It leaks from the cerebrospinal fluid into blood after cerebral damage and is an indicator of brain injury as well as increased permeability of the blood brain barrier. The determination of S100β concentrations in serum is performed with an ELISA. It can be detected in 50 µl samples of serum, heparin plasma or urine.

1.4.5 Neurological developmental assessment tool

Neurodevelopmental outcome should be evaluated by standardised tests which are psychometric measures designed to inventory an infant's skills against a 'normal' population within a specific assessment. The standardisation is designed to reduce measurement error by precluding subjective interpretation of the child's responses. Bayley Scales of Infant Development (BSID) is such a standardised test (21). BSID was revised and re-standardised in 1993 to form the BSID-II and in 2005 to form the BSD-III and is widely used worldwide as outcome measure in clinical and research practice. BSID-III provides a multi-domain assessment of children aged 1 to 42 months. It reveals five major developmental domains: cognitive, language, motor, adaptive behaviour, and social-emotional. The scale also includes a parent report form, the Adaptive Behaviour Questionnaires that provides scores based on parents' perceptions of the child's level of function. In this trial, the BSID-III will be performed at 24 months after term date, using the cognitive, language, and motor domains.

The Ages & Stages Questionnaire (ASQ) is a parental survey that comprises questionnaires for children age 4 months to 5 years. It consists of 6 questions in each of 5 developmental domains covering motor as well as mental development. The agreement between ASQ and with concurrent assessment by BSID-III is good and ASQ can be a substitute for the BSID-III in cases or at centres where the BSID-III is not possible (22).

1.5 Cerebral oximetry monitoring

SafeBoosC phase II

Near-infrared spectroscopy (NIRS) is a non-invasive technology that has been utilised to assess the adequacy of peripheral and cerebral oxygenation in the preterm infant (23). Near-infrared light penetrates deep into the tissue, and through spectroscopy, it is possible to monitor tissue oxygenation. NIRS uses the relative transparency of human tissue to light in the near-infrared region of the spectrum. The oxygen-dependent absorption of light by haemoglobin enables the calculation of relative changes in the oxygenated and deoxygenated haemoglobin (24). The NIRS has been used in newborns since 1985 (25) and it is particularly suitable for the neonatal population due to their thin scalp and skull. Newer generations of oximeters provide an absolute value of tissue oxygenation (rStO2). This is most often done by spatially resolved spectroscopy. The assumption behind is that light propagates in a diffusional manner in a highly scattering media such as human tissue, and that the light attenuation by scattering is constant with light distances over 3 centimetres. The rStO2 can then be expressed as (k x O2HB)/(k x O2HB + k x HHB), where k is the scattering component which cancels out (26). Examples of some of the commercialised NIRS devices are INVOS® System, NIRO Series, and Casmed Foresight.

NIRS is based on the same principles as the widely used pulse oximetry, but where pulse oximetry uses the pulsating signal and thereby selectively measures arterial blood, NIRS measures the light attenuation of the tissue as a whole. This means that venous blood contributes more to the attenuation than arterial blood simply because venous blood has a greater volume. The ratio of venous:arterial contribution is generally considered to be 75:25, although this has been found to differ between and within infants (27). It is thus not surprising that cerebral tissue oxygenation has shown a fair correlation with the saturation in cerebral venous blood drawn from the jugular bulb (28). The Bland-Altman limit of agreement is ± 15-

20% (29,30). It has to be remembered that rStO2 is volume weighted, whereas jugular bulb saturation is flow weighted. Good agreement cannot be expected. It could be that during conditions where the microcirculation is compromised such as sepsis, rStO2 may give a better picture of the cerebral oxygen balance than jugular bulb saturation (31).

1.5.1 NIRS devices

Different NIRS devices differ in absolute values and in dynamic ranges (32).There is no 'reference standard' for tissue oximetry. Therefore, comparison of devices can be done in a standardised setup in the forearm of healthy adults. Reproducibility is assessed by re-siting of the sensors during steady state and a test of dynamic range is done by arm exercise and subsequent arterial occlusion by a cuff. Four devices have been compared in a preliminary study:

- NIRO 200 NX;
- NIRO 300;
- INVOS 5100c; and
- OxyPrem (a prototype).

The reproducibility of the NIRO 200 NX, NIRO 300, and the INVOS 5100c was similar (within-subject standard deviation 4.35%, 4.10% and 5.46%, respectively). In addition, these devices showed similar absolute oxygenation (rStO2) values (63% to 70%) and similar dynamic range. The OxyPrem showed significantly better reproducibility of 2.7%, but a somewhat lower absolute oxygenation (rStO2) value (60%) and dynamic range only two-thirds of the other three instruments (33) The results are comparable to earlier studies on the head regarding reproducibility and inter-instrument differences (32,34-37)

SafeBoosC phase II

All eligible candidate NIRS devices for use in the SafeBoosC trial will be tested by the same procedure and should be within five percentage points in mean values and dynamic range in comparison with the above devices, and have a reproducibility of 6% or better. NIRS devices and sensors are non-sterile.

The SafeBoosC trial is a clinical investigation of benefits and harms of a range of different NIRS devices in combination with a treatment guideline. The trial is not designed to assess the performance of each NIRS device, since no reference standard is available in the preterm population. It will however evaluate whether each NIRS device is suitable for the trial population by careful collection of all adverse device effects.

Each participating centre is required to provide NIRS device(s) for the SafeBoosC trial. Each country shall apply with the regulations regarding the use of their device(s) in a clinical trial (e.g., a CE-mark that indicates use for the SafeBoosC trial population, as laid down by the national competent authority).

The investigational NIRS device at each participating trial site is identified and described, including instructions for installation and use, in the site-specific investigator's brochure (IB). All material in the IB are provided by the sponsor. The investigator at each trial site is responsible

for education and training in cerebral NIRS oximetry and the relevant NIRS device. The required training before use of each device is described in the relevant IB.

1.6 Regional oxygenation saturation in preterm infants

A study conducted by McNeil *et al*, 2011, characterises the baseline cerebral rStO2 in 12 stable preterm infants (gestational age (GA) 29-34 weeks) during the first weeks of life to be between 66-83% (38). In the study conducted by Zhou *et al*., 2009, where they enrolled a total of 223 full-term newborns, the 'normal' cerebral rStO2 was determined to be between 62 ± 2% and cerebral hypoxia was defined as rStO2 less than 58% (39). Derived from data, collected over time, from about 390 preterm babies (born at GA<32wks) during the first 3 days of life, van Bel *et al*. (unpublished data) concluded the rStO2 baseline target range in this population to be 55-85% (±2SD) with the mean value of 71%. The data was collected with the INVOS 4100/5100 with the Adult Somasenor® are constitutes the basis of the normal ranges in SafeBoosC.

1.7 Previous trials on similar research question

There is a variety of observational studies that document, that cerebral oximetry in preterm infants gives meaningful physiological data. Wolf and Greisen systematically reviewed 36 studies in neonates that all contribute to an understanding of oxygen delivery-consumption balance in this population (24). Based on their study, however, we are able to conclude that evidence of the clinical benefits and harms of cerebral oximetry in preterm infants does not exist.

SafeBoosC phase II

We systematically searched in four medical literature databases on November 29, 2011: Cochrane Library, MEDLINE, EMBASE, and Science Citation Index Expanded. Using the keywords 'near-infrared spectroscopy', 'NIRS' 'preterm', 'infant', 'neonatal' and 'newborn', the searches yielded a total 1, 797, 534, and 372 hits, respectively. The search found no published randomised clinical trials (RCTs) focusing on the effect of cerebral monitoring in preterm infants in combination of clinical treatment guidelines based on the rStO2 produced by the NIRS oximeter.

A search on ClinicalTrials.gov (www.ClinicialTrials.gov) on November 29, 2011 using the keyword 'NIRS' and 'Near Infrared spectroscopy' on age group birth to 17 years gave 40 and 44 hits, respectively. Only one trial on preterm infants, Treatment of Hypotension of Prematurity (TOHOP) (NCT01434251), involves cerebral NIRS oximetry as part of the trial intervention. NIRS is part of an assessment of tissue perfusion and end-organ function. If StO2 is normal a low blood pressure will be tolerated in the intervention group. It is thus not the benefits of NIRS that are evaluated, but the possible benefits of allowing a low blood pressure when the tissue perfusion is not compromised. The NIRS readings will be visible in both groups. The trial is currently recruiting participants (Sept. 2011).

Hence, no study investigates the possible benefits and harms of cerebral NIRS oximetry out-of-range readings as an indication to intervene in the preterm infant.

The evidence of cerebral oximetry in adults is also limited. To date, there is only one published systematic review of evidence of clinical utility of cerebral oximetry in adults during coronary surgery concluding that, with data from 47 trials including more than 5,000 participants, the methodological quality of the trials was low and therefore clinical benefits and harms are uncertain (40).

1.8 Trial rationale

There is accumulating evidence that hypoxia and hyperoxia is associated with the risk of brain injury and death in prematurely born infants. Thus, the monitoring of cerebral oxygen saturation levels in the first hours after birth and subsequent treatment according to pre-specified guidelines has a great potential for prevention of brain injury and maybe death. Despite this, there are no randomised clinical trials on the benefit and harms of NIRS in preterm infants. Yet, the technology is increasingly implemented in clinical care. To obtain evidence-based knowledge on the benefits and harms of cerebral monitoring using NIRS as a part of clinical management of premature infants, a large-scale RCT is needed.

1.9 Justification for the design of the clinical investigation

This clinical investigation of NIRS devices is investigator initiated and funded on public donations. It will include several different investigational devices. The different device manufacturers have not been involved in any part of the design or funding of the investigation. All pre-clinical and clinical data are from published studies. Clinical evaluation of each specific investigational device is provided in the site-specific IB.

SafeBoosC phase II

1.9.1 Rationale for use of INVOS Adult SomaSensors® as reference standard for mean values in neonates in the study

This clinical investigation will use the INVOS 5100c with the adult SomaSensors® as reference standard for NIRS device eligibility, because the normal material of 390 infants that defines the target thresholds of $rStO_{2,}$ 55 and 85%, comes from the group of Petra Lemmers and Frank Van Bel using that sensor. The application of the Adult SomaSensor in this population is considered safe as no serious adverse device effects, such as skin burns etc., were encountered during the monitoring of these infants (41-45). Data collected with the Adult SomaSensor indicates that a high $rStO_2$ could possibly predict poor outcome (46). Clinical data suggests that the INVOS 5100c neonatal sensor OxyAlert™ NIRSensors gives values that are about 10 percentage points higher that what found with the Adult SomaSensors® and NIRO 300 (32,47,48). This means that $rStO_2$ above 85% as defined by the adult SomaSensors® cannot be monitored with the OxyAlert™ NIRSensors as the device cannot give values above 95%,, values that moreover are physiologically unreasonable. As described above other device-sensor combinations are eligible provided fulfilment of the specified criteria.

1.9.2 Rationale for the evaluation of the investigational devices

Cerebral oximetry by NIRS in extremely preterm infants has no reference standard, and a study of device performance in this population is thus no possible. The design of this clinical

investigation is targeted at assessment of the clinical utility of NIRS by assessing whether $rStO_2$ can be kept within normal ranges by pre-specified medical interventions. Whether each investigational device (CE or non-CE marked) is suitable for the trial population, will be assessed be careful collection of all adverse device effects.

2. Trial objective and hypothesis

The objectives of this phase II trial are to:

- examine whether it is possible to reduce the burden of cerebral hypoxia and hyperoxia through the application of NIRS and the implementation of a set of defined clinical treatment guidelines.
- evaluate the feasibility of a large-scale randomised clinical trial of complex interventions in European neonatal units;
- explore the relationships between cerebral hypo- and hyperoxia, clinical interventions, and markers of brain injury in extremely preterm infants.

We hypothesise that by using a specified treatment guideline to cerebral monitoring readings outside the target range of 55-85% we would reduce the burden of cerebral hypo- and hyperoxia in order to reduce brain injury in preterm infants.

SafeBoosC phase II

3. Trial design

This is an investigator-initiated randomised, blinded, multinational, phase II feasibility clinical trial that will enrol 166 preterm infants from European countries (Figure 1).

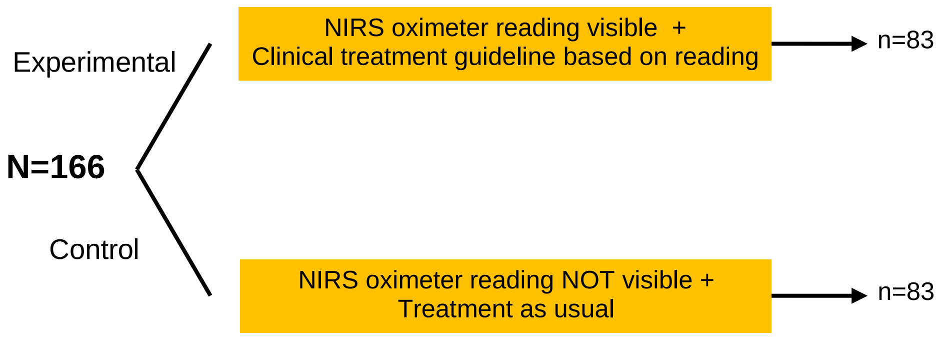

Figure 1: Trial design

3.1 Randomisation

Participants will be randomised into either the experimental group or the control group. The allocation sequence will be computer-generated with a varying block size, and is kept concealed for all investigators. The ratio of allocation is 1:1. The randomisation will be stratified by the variable gestational age (low gestational age (<26 weeks) vs. high gestational age (≥26 weeks)). Randomisation will be centralised at the Copenhagen Trial Unit and web-based.

Singleton infants will be randomised individually. Multiple birth infants will be randomised as a ‘pair’ or a ‘group’, i.e., all siblings will be allocated to the same treatment group. In centres, where only one or two cerebral monitoring devices are available, it may not be possible to include all infants from multiple-births, Thus, only one of a pair will be included or eventually only one or two if triplets. The sibling(s) enrolled will be the one(s) born last.

3.2 Trial interventions

The premature infants will be randomised into one of the two groups. Common is that both the experimental and the control group will have a cerebral NIRS oximeter monitoring device placed within three hours after birth.

- *Experimental:* The cerebral oxygenation reading is visible, and the infant will be treated according to a defined treatment guideline (see appendix A).
- *Control:* The cerebral oxygenation reading is NOT visible, and the infant will be treated according to standard clinical practice (‘treatment as usual).

SafeBoosC phase II

3.3 Duration

Cerebral monitoring will start within three hours of age and the intervention will last until 72 hours after birth, as these are the most critical. Each neonate will be followed up at the term date (approximately after 3 months) and 24 months after the term date (Figure 2).

Trial timeline

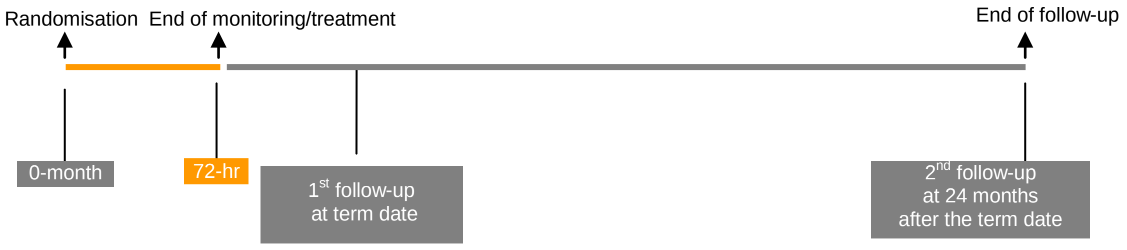

Figure 2: SafeBoosC Trial timeline

3.4 Blinding

Due to the nature of the intervention, the intervention cannot be blinded for the clinical staff and the parents. However, blinding will be used in all other aspects of the trial. Firstly, the allocation sequence will be concealed for the investigators and all other trial personnel. Secondly, some

outcomes, including the primary, will be assessed by an outcome assessment committee (centrally), blinded to the allocation of the participants. Thirdly, the statistical data analyses will be performed with the two intervention groups concealed as, e.g., X and Y. Finally, two conclusions of the results of the trial will be drawn – one presuming X is the experimental group and Y the control, and one assuming the opposite. Hereafter, the blinding will be broken.

4. Participants

4.1 Inclusion criteria

Neonates meeting the following criteria will be included:

- Neonates born more than 12 weeks preterm (gestational age up to 27 weeks and 6 days).
- Decision to conduct full life support.
- Possibility to place cerebral NIRS oximeter within 3 hours after birth.
- Obtained parental signed written informed consent.

4.2 Exclusion criteria

Neonates meeting the following criteria will be excluded:

- A clinical decision not to provide full life support.
- No possibility to place the cerebral NIRS oximeter within 3 hours after birth.
- Lack of parental signed written informed consent.

SafeBoosC phase II

4.3 Exclusion Period

There will be no exclusion period.

Neonates included in the SafeBoosC trial can participate in any other intervention study duringthe first 72 hours, on the condition that the study does not:

a) use visible cerebral oximetry in the control group, or
b) exclude a treatment that would be clearly indicated by the SafeBoosC treatment guideline

After the first 72h there will be no restrictions.

4.4 Participant discontinuation and withdrawal

The participant's parents are free to withdraw the participant from the intervention or from the SafeBoosC trial entirely at any time, and this will not have any consequences for the participant's further treatment. The reason(s) will be recorded. When possible, the parents will be asked if they will allow their child to participate in the remaining follow-up assessments, and allow their child's already collected data to be used in a database, a registry, and/or a publication. If withdrawal is due to problems related to the investigational device the parent's will be asked for permission to follow the status/condition outside the clinical investigation. The follow-up will be individualised.

The attending clinician can withdraw the participant from the trial at any time. The reasons shall be documented. There are no pre-specified criteria for discontinuation of participants from trial.

The discontinuation of participants in the trial will not result in replacement with new participants.

4.5 Recruitment feasibility

Neonates are expected to be included from 12 neonatal intensive care units (NICUs) in 12 European countries. The units have estimated the likely rate of recruitment between 10 and 40 infants per year. The total is about 145-310 infants per year. The trial should therefore have good chance to be able to recruit 166 participants within a year. There are no defined minimum or maximum number of subjects to be included in each centre, since this is a phase 2 trial not powered to detect clinical outcome differences, but well-powered to detect the minimal clinical significant difference in $rStO_2$.

5. Interventions

5.1 Common to both groups

Both groups will have a cerebral NIRS oximeter placed within three hours after birth. Cerebral oximeters meeting the following validity and reliability criteria will be allowed in the trial: precision better than 6%, accuracy as well as dynamic range within 5% points, sensor type appropriate for the oximeters meeting the aforementioned criteria. In centres that have more than one NIRS device, a ranking list shall be designed and applied.

SafeBoosC phase II

5.2 Experimental group

In the experimental group, the reading of the cerebral oxygenation will be visible. A clinical treatment guideline recommending adjustments of respiratory and cardiovascular support will be followed to keep cerebral oxygenation within defined target range of 55% to 85%. The treatment guideline is detailed in Appendix A and will be used for all participants allocated to the experimental group regardless of countries where the treatment is given.

5.3 Control group

In the control group, the reading of the cerebral oxygenation will NOT be visible. The infants will be given the best standard treatment – ‘treatment us usual’.

A locked box will be used to cover the cerebral NIRS oximeter used in the control group to ensure that the reading of the oximeter is not visible to the physician. The signal quality indicator of the cerebral oximeter will be visible to allow the clinical staff to replace the sensor as needed.

5.4 Concomitant medication/treatment

There is no specified ‘per-protocol’ concomitant medication or treatment, as any other aspects of treatment not defined in the treatment guideline is per choice of the treating physician and the treating team.

5.5 Medical care after end of clinical investigation

It is unlikely that the trial participants will need medical care at the end of this clinical investigation at 2 years corrected age. If needed, it will be according to local guidelines.

6. Outcome measures

The outcome measures are listed below. Please see more details in table 1 and in appendices B-E.

6.1 Primary

The primary outcome is:

- Burden of hypo- and hyperoxia in %hours during the first 72 hours after birth.

6.2 Secondary

The secondary outcomes are:

- Brain activities on aEEG as assessed by the interburst interval.
- Brain injury by cerebral ultrasound.
- All-cause mortality.

SafeBoosC phase II

6.3 Exploratory

The exploratory outcomes are:

- Blood and urine biomarkers (brain fatty acid binding protein, neuroketal, and S100β).
- Serious adverse reactions.
- Burden of hypoxia.
- Burden of hyperoxia.
- Neonatal morbidities (necrotizing enterocolitis (NEC) stage 2-3, bronchopulmonary dysplasia (BPD) defined as oxygen requirement at 36 weeks, and retinopathy of prematurity (ROP) stage 3+ and above).
- aEEG band power and EEG patterns.
- Brain injury score according to Woodward and quantitative measurements of brain maturity and brain volumes on MRI.
- Number of therapies implemented during the intervention (mechanical ventilation, volume substitution, blood transfusion, inotrope, vasopressor, ductal closure).
- Physiological parameters (mean BP, SpO_2, and pCO_2)
- BSID-III (cognitive score, verbal score, and motor score).
- ASQ (score between 0-60 from 5 domains).

6.4 Outcome assessment tools

The outcome assessment tools used in the trials are the aEEG, cUS, biomarkers, MRI, and the psychomotor scales (BSID-III and ASQ). Table 1 lists the outcome measures to be assessed and the time points of the assessments.

6.5 Outcome assessment committee

The reading and interpretation of the outcomes will be centralised and the assessor will be blinded of the participants' intervention group (see table 1).

6.6 Compliance with the CIP

The clinical investigation will be conducted in compliance with this CIP. Any appropriate modifications will be discussed in the trial steering committee. Modifications to the CIP will not be implemented before agreement from the sponsor, relevant ethics committee, and regulatory authorities are obtained.

Investigators are not allowed to deviate from the CIP except as specified above. Any major or safety related deviations will be recorded, analysed and reported to the regulatory authorities and ECs within 7 workdays. If an investigator refuses to comply with the CIP he/she will be disqualified.

7. Data collection and trial assessment schedule

SafeBoosC phase II

7.1 Collection of trial data

7.1.1 NIRS device

Data of cerebral oxygenation (burden of hypoxia and hyperoxia) are collected directly from the NIRS device. Each site will download NIRS data to the data management centre after completion of the participant's intervention period at 72 hours after birth. A specialised software program, developed for the purpose, will be used.

7.1.2 Case record form

Other trial data will be collected using an electronic case record form (eCRF) as primary entry point. The CRFs will be designed in collaboration between the Data Manager at the Copenhagen Trial Unit and the sponsor (see section 11.1).

Table 1: Assessment tools and outcome measures

Tools	Outcomes	Time points*	Centralised reading#
Cerebral NIRS oximeter	• Burden of hypoxia<br>• Burden of hyperoxia	• During the intervention until 72 hours after birth	Department of Neonatology, Rigshospitalet, Denmark
aEEG/EEG (Appendix C)	• Interburst interval (IBI)<br>• Power in delta band<br>• Power in theta band<br>• Power in alpha band<br>• Power in beta band	• 64 hours after birth	Uppsala University, Sweden

Tools	Outcomes	Time points*	Centralised reading[#]
Biomarkers (Appendix B)	• BFBP<br>• Neuroketal<br>• S100β	• 6 hours after birth<br>• 64 hours birth	Haemoscan B.V., The Netherlands
Investigator's assessment and medical records	• Serious adverse reactions<br>• Non-serious adverse reactions	• During the intervention until 72 hours after birth	Not centralised
cUS (Appendix E)	• IVH grade (Papille)<br>• Cerebellar haemorrhage<br>• cPVL<br>• Cerebral atrophy<br>• Post-haemorrhagic hydrocephalus	During the intervention and follow-up period, according to these assessment points:<br>• At 1day after birth<br>• At 4 days after birth<br>• At 7 days after birth<br>• At 14 days after birth<br>• At 35 days after birth<br>• At 40-44 weeks	Department of Neonatology, Wilhelmina Children's hospital, UMC Utrecht, The Netherlands
Investigator's assessment and medical records	• All-cause mortality	• Term date<br>• 24 months after term date	Not centralised
Investigator's assessment and medical records	Neonatal morbidities:<br>• NEC stage 2-3<br>• ROP stage 3+ and above	• Term date	Not centralised
Investigator's assessment and medical records	Neonatal morbidities:<br>• Oxygen requirement	• At 36 weeks	Not centralised
MRI (Appendix D)	• Brain injury score (Woodward)<br>• Volumetric<br>• Cortical folding Diffusion tensor imaging	• At term date (approximately three months after birth)	Department of Neonatology, Wilhelmina Children's hospital, UMC Utrecht, The Netherlands
Investigator's assessment and medical records	Therapies implemented:<br>• Mechanical ventilation<br>• Volume substitution<br>• Blood transfusion<br>• Inotrope<br>• Vasopressor<br>• Ductal closure	• During the intervention until 72 hours after birth	Not centralised
CRF	Physiological parameters<br>• Mean BP<br>• Mean SpO2<br>• Mean pCO2	• During the intervention until 72 hours after birth	Not centralised
BSID-III	• Cognitive score<br>• Verbal score<br>• Motor score	• 24 months after term date	Not centralised
ASQ	• Communication<br>• Gross motor<br>• Fine motor<br>• Problem solving<br>• Personal-social	• 24 months after term date	Not centralised

* If assessment is not possible at the specified time, the assessment shall still be conducted, and the time will appear in the eCRF and the deviations from the CIP can be assessed.
[#] Central assessment is conducted by a blinded outcome assessment committee.

SafeBoosC phase II

7.2 Trial assessment schedule

Trial data to be collected and the specified time points are detailed in Table 2 - Trial assessment schedule.

7.3 Research biobank

Blood and urine samples will be stored at the individual clinical sites according to national regulations and guidelines and used for assessment of biomarkers BFABP, neuroketal, and S100β.

Blood

If a central vascular catheter is in place up to one ml of blood will be collected in **heparin**. After processing for obtaining serum this must be stored frozen (-20ºC ±4ºC for a maximum of one week, thereafter at -80ºC ±10ºC for prolonged storage). Blood will be drawn 2 times, and in total 1 ml serum will be stored per participant.

Urine

Urine will be collected only if a urinary catheter is already in place or can be done as part of routine non-invasive urine collection.1 ml of urine shall be stored frozen (-20ºC ±4ºC for a maximum of one week, thereafter at -80ºC ±10ºC for prolonged storage) without further treatment. Urine samples will be collected 2 times, and in total 2 ml will be stored per participant. There are no immediate risks with obtaining the urine samples.

Storing

Serum and urine samples are labelled with unique identification (as outlined in appendix B). Each principal investigator shall keep record of the participant identity linked to the participant number. All samples should be stored locally at the site until last participants samples has been collected, then all samples will be shipped to Haemoscan, Netherlands (refer to appendix B). After analysis - expected to be in 2014 - all remaining samples after final analysis will be discarded.

SafeBoosC phase II

Table 2: Trial assessment schedule

**Visit Number**	**0**	**1**	**2**				**3**	**4**	**5**	**6**	**7**	**8**
**Visit description**	**Screening**	**Randomisatio n**	**Intervention period (Cerebral monitoring ± visibility and treatment according to guideline or 'us usual')**					**Follow-up**				
**Visit code**	**V0**	**V1**	**V2a**	**V2b**	**V2c**	**V2d**	**V3**	**V4**	**V5**	**V6**	**V7**	**V8**
**Time period**	**0-hour**	**0-3 hours after birth**	**3 hours after birth**	**6 hours after birth**	**24 hours after birth**	**64 hours after birth**	**4 days after birth**	**7 days after birth**	**14 days after birth**	**35 days after birth**	**Term date**	**24 months after term date**
**Informed consent** can be obtained before birth	X											
**In- and exclusion criteria**	X											
**Maternal history**	X											
**Maternal antenatal concomitant medication**	X											
**Infant concomitant medication**	X		X	X	X	X						
**Placement of cerebral NIRS oximeter** between 0-3 hours after birth		X										
**Blood/urine (biomarker)**				X		X						
**Serious adverse reaction**			X	X	X	X		X				
**Non-serious adverse reactions**			X	X	X	X						
**Neonatal comorbidities**											X	
**aEEG**						X						
**cUS**					X		X	X	X	X	X	
**MRI**											X	
**BSID-III**												X
**ASQ**												X
**All-cause mortality**											X	X

SafeBoosC phase II

8. Assessment of safety

8.1 Adverse events and reactions

8.1.1 Definitions

Adverse events (AE): any undesirable event occurring to a participant during a clinical trial, whether or not considered related to the trial intervention.

Serious adverse event (SAE): any adverse event that results in death, is life-threatening, requires prolongation of existing hospitalisation, result in persistent or significant disability or incapacity, or requires intervention to prevent permanent impairment or damage.

Adverse reactions (AR) / Adverse device effects (ADE): all untoward and unintended responses related to the interventions of application of the treatment guideline and/or cerebral oximeter.

Expected adverse reactions (EAR) / Expected adverse device effect (EADE): adverse reactions, we expect to be related to the interventions cerebral NIRS oximeter and/or the application of the treatment guideline are:

SafeBoosC phase II

- Local skin reactions (rashes, burns, etc.).
- Reactions related to the manipulation of the patient during positing and re-positing of the cerebral NIRS oximeter sensors.
    - Accidental displacements of the endotracheal tube or extubation.
    - Accidental displacement of venous or arterial catheters,
    - Severe hypoxia or bradycardia otherwise unexplained.
- Other expected adverse reactions encompass reactions to any interventions directed at correcting an out-of-range oximeter reading by improving respiratory status, cardiovascular status, oxygen transport, and blood glucose level (see appendix A).

Unexpected adverse reactions (UAR): all untoward and unintended responses, that not is an expected reaction, related to the interventions cerebral oximeter and/or application of the treatment guideline.

Serious adverse reactions (SAR): any adverse reaction that results in death, is life-threatening, requires prolongation of existing hospitalisation, results in persistent or significant disability or incapacity, or requires intervention to prevent permanent impairment or damage. This definition includes **Serious adverse device effect (SADE)**: adverse device effect that has resulted in any of the consequences characteristic of a SAE, or are due to

imprecise or incomplete results from diagnostic equipment e.g. incorrect or delayed diagnosis or incorrect or delayed treatment and **Serious adverse effect-near-incidents (SAE-NI):** device deficiencies that might have led to a SAE if a suitable action had not been taken, or intervention had not been made, or if circumstances had been less fortunate.

Expected serious adverse reactions (ESAR): any of the expected adverse reactions (listed above) that results in death, is life-threatening, requires prolongation of existing hospitalisation, results in persistent or significant disability or incapacity, or requires intervention to prevent permanent impairment or damage.

Suspected unexpected serious adverse reaction (SUSAR) / Unanticipated serious adverse device effect (USADE): an adverse reaction which is both serious and unexpected, i.e., not identified in the current risk analysis listed above (in expected adverse reactions). Included in this definition are the events with imminent risk of death, serious injury, or serious illness that requires prompt remedial action for other participants, users, or persons (including events that are of significant and unexpected nature such that they become alarming as a potential public health hazard or the possibility of multiple deaths occurring at short intervals) or a new finding to the event.

SafeBoosC phase II

Device deficiencies: malfunction, misuse, or use error, e.g. $rStO_2$ values displayed despite displaced sensor, sensor positioned with light source facing away from skin surface, sudden malfunction of device that result in no or false $rStO_2$-values.

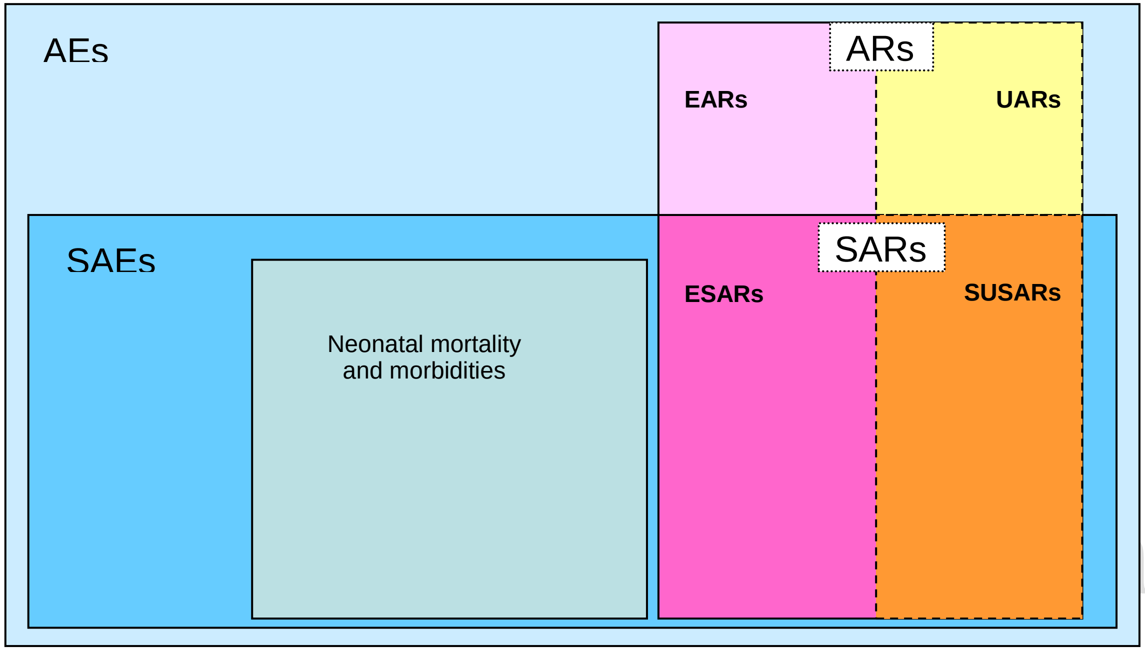

ase II

Figure 3: Relation among adverse events and reactions

8.1.2 Classification of causality

Classification of causality of adverse reactions are outlined in table 3.

8.1.3 Recording and reporting of adverse events and reactions and device deficiencies

Adverse events and device deficiencies are recorded and reported to the competent authority in all countries in which the trial is being conducted according to national guidelines, Directive 90/42/ECC, as amended by the Directive 2007/47/EC and the EU Commission guidelines on medical devices, Meddev 2.7/3 "Clinical investigations: serious adverse event reporting".

A different periodicity and modality will be implemented (refer to section 8.1.4) in accordance with the EU Commission guidelines on medical devices, Meddev 2.7/3 "Clinical investigations: serious adverse event reporting", and needs to be agreed between sponsor and the relevant national competent authorities.

An overview of the recording and reporting of adverse events is found in table 4.

Table 3: Classification of causality of adverse reactions

**Certain**	Event or laboratory test abnormality, with plausible time relationship to the SafeBoosC trial interventions; cannot be explained by disease or other treatments; response to withdrawal is clinically plausible (pharmacologically, pathologically); event definitive pharmacologically or phenomenologically (i.e., an objective and specific medical disorder or a recognised pharmacological phenomenon); or re-challenge satisfactory, if necessary.
**Probable / likely**	Event or laboratory test abnormality, with reasonable time relationship to the SafeBoosC trial interventions; unlikely to be attributed to disease or other treatments; response to withdrawal clinically reasonable; re-challenge not required.
**Possible**	Event or laboratory test abnormality, with reasonable time relationship to the SafeBoosC trial interventions; could also be explained by disease or other treatments; information on intervention withdrawal may be lacking or unclear.
**Unlikely**	Event or laboratory test abnormality, with a time to the SafeBoosC trial interventions that makes a relationship improbable (but not impossible); disease or other treatments provide plausible explanations.
**Conditional / unclassified**	Event or laboratory test abnormality; more data for proper assessment needed; or additional data under examination.
**Unassessable / unclassifiable**	Report suggesting an adverse reaction; cannot be judged because information is insufficient or contradictory; data cannot be supplemented or verified.

SafeBoosC phase II

Table 4: Recording and reporting of adverse events and reactions

Type of event	Recorded	Reported as an outcome measure	Reported** during the trial	Reported** yearly	Reported** end of trial
AEs	-	-	-	-	-
SAEs	-	-	-	-	-
Neonatal mortality and morbidities	yes	Yes	-	-	-
EARs*	yes	yes	-	-	yes
UARs*	yes	yes	-	-	yes
ESARs*	yes	Yes	-	Yes	Yes
SUSARs*	yes	Yes	Yes	Yes	Yes

*Adverse reactions with a 'certain' or 'probable/likely' relation to the intervention

**** Reported to the relevant national competent authorities**

SafeBoosC phase II

8.1.4 Justification for recording and reporting periodicity and modality

The preterm patient population is a very seriously ill group. Most adverse events may be of a serious nature with or without the SafeBoosC trial intervention, and both intervention groups are expected to have a very high proportion of serious adverse events. It is therefore not possible, nor meaningful to record and report all adverse events. The following will be recorded and reported (for overview refer to table 4):

Adverse reactions (i.e. both expected (EAR) and unexpected (UAR) with a 'certain' or 'probable/likely' relation (see table 3) to the interventions will be recorded together with an assessment and reported with the trial results, The final report clinical investigation report to the competent authorities will summarize the ARs, including a discussion of causal relationship with investigational device, severity, and treatment needed.

Serious adverse events (SAE) to be recorded are: mortality; the mortality is high and will thus be analysed and reported as a secondary outcome measure and thus will not be reported to the authorities, and neonatal morbidities; these are believed to be the most important predictors for later development and health, and are recorded and reported as an outcome measure (necrotising enterocolitis stage 2-3, oxygen requirement, and

retinopathy of prematurity stage 3+ and above, as well as severe intraventricular haemorrhage and periventricular leucomalacia) thus not reported to the competent authorities.

Serious adverse reactions (i.e., both expected (ESAR) and unexpected (SUSAR) related to the cerebral oximeter and/or the treatment guideline will be recorded and analysed as a secondary outcome measure, but only SUSARs will subject to expedited reporting. Due to the nature of the population and the intervention, the near-incidents (**SAE-NI)** will be recorded, but not reported to the authorities.

8.1.5 Timelines for recording and reporting

The site investigators will report the SAEs (mortality and neonatal morbidities) and the EARs and UARs through the eCRF periodically according to the assessment schedule (refer to section 7.2).

The site investigators will report all SARs (ESARs and SUSARs) and device deficiencies with potential severe effects to the sponsor through the eCRF or by fax if necessary and by phone (+4535451326) immediately and within 24 hours of knowledge. The ESARs, or new information regarding already reported events will be reported by the sponsor to the relevant competent authorities in the yearly report. The sponsor will immediately inform all investigators of any SUSAR as soon as s/he is made aware of the event. The sponsor will submit an expedited report of all SUSARs or new information in relation with an already reported event, to the relevant competent authorities within 2 calendar days of knowledge. The sponsor will review all SUSARs. If the judgement of sponsor differs from the investigator, both assessments of the SUSAR shall be included in the SUSAR report to the authorities.

SafeBoosC phase II

As the trial is estimated to be finalised within one year, the annual report will be omitted and replaced by the end of trial report in the case where the reporting timelines are overlapping with 3 months.

8.2 Cerebral NIRS monitoring device

A summary of the risk analysis for each investigational NIRS device is provided in the relevant IB. To ensure safe application of the device investigators and the involved staff will be trained on how to place the device (see section 9.2). For extended monitoring the sensor should be repositioned at a different location, as often as judged necessary. For infants with scalp oedema, or poor perfusion this may be as often as every 4 hours in order to avoid damage from heat and/or pressure. Optimally, replacement should be done as part of the routine handling of the infant to disturb the infant as little as possible.

Further, if skin irritation does occur, it will be treated according to local clinical practice. Interaction with the drugs applied in this population is not possible.

The requirements specified in ISO14155:2011 "6.9 Investigational device accountability" does not apply to this clinical investigation. All devices are owned by the local institution and not provided by the sponsor.

8.3 Data Monitoring and Safety Committee

An independent Data Monitoring and Safety Committee (DMSC) is established to, during the trial, monitor SUSARs with ‘certain’ or ‘probably/likely’ relationship with the cerebral NIRS oximeter or the application of the treatment guideline. The charter for the DMSC will be written prior to inclusion of participants and prior to any analysis. The members of the DSMC are listed under “Data Monitoring and Safety Committee” on p. 9.

8.4 Suspension or premature termination of the clinical investigation

The sponsor/principal investigator, the ECs, and the regulatory authorities can decide about trial continuation. The clinical investigation can be suspended or prematurely terminated if the serious adverse device effects are considered disproportionately large compared to the possible benefits of the intervention. If the trial is terminated or suspended the parents of all trial participants will be informed and appropriate follow-up will be assured. If sponsor/principal investigator terminates or suspends the trial the relevant ECs and regulatory authorities will be provided with a detailed written explanation of the termination or suspension.

SafeBoosC phase II

The sponsor/principal investigator can upon completion of the analysis of the reason(s) for a suspension decide to lift the suspension, when the necessary corrective actions have been implemented. The investigators, ECs, and relevant regulatory authorities will be notified and provided with the relevant data supporting the decision.

Breaking of blinding will not be relevant in this trial, since group allocation is visible and the assessment of the adverse device effects in the control group are independent of $rStO_2$ readings of the device.

9. Ethical Considerations

The research question; can monitoring of cerebral oxygen saturation levels in the first hours after birth and subsequent treatment according to pre-specified guidelines prevent brain injury and maybe death, can only be answered in the specified population, and there is a clear benefit to the premature patient population as a whole, should the research gain a positive outcome (research in the Research Ethics Committee group c) As there are no randomised clinical trials (RCT), thus no conclusive evidence, on the benefit and harms of NIRS monitoring in preterm infants, we find it ethically suitable that the control group receives 'treatment as usual'. All interventions proposed in the treatment guideline are commonly used in this patient group.

There is clinical equipoise, which means that there is genuine uncertainty over whether the cerebral oximeter and subsequent treatment will be beneficial or may even be harmful to the participants. Due to the clinical nature of the trial population, the premature infants, some stress reactions related to the manipulation during positing and re-positing of the cerebral NIRS oximeter sensors can occur. These are however not clinically evaluated to give substantially more risk or discomfort as compared to no intervention. To obtain evidence-based knowledge on the benefit and harms of cerebral monitoring using NIRS as part of clinical management of premature infants, a large-scale RCT is needed. The SafeBoosC phase II trial serves as a feasibility trial for such a large phase III trial.

SafeBoosC phase II

The SafeBoosC trial will only start the randomisation of participants after approvals from the relevant ethics committees and competent authorities have been received and parental informed consent is obtained. All parents will receive written and oral information about the trial before they are asked for their written consent (se section 9.2 for further details). They will only enrol their new-born in the trial by their own free will and can withdraw their consent for participation at any time. If a parent wishes to withdraw the participation, the patient will be treated according to the respective hospital's standard procedures. The trial will be conducted in compliance with the guidelines of the Declaration of Helsinki in its latest form and the International Conference on Harmonization of Good Clinical Practice Guidelines (49) To avoid concern it has been decided that multiple births will be randomised together and undergo allocation to the same intervention.

9.1 Informed consent procedure

Parents of potential participants will be invited to enrol their preterm newborn, when possible, before delivery. Due to the nature of the intervention, if potential participants are found after their delivery, informed consent procedures must take place within 3 hrs. Surrogate informed consent is required

from both parents, unless one parent has power of attorney. The sponsor/sponsor delegate (qualified physician or nurse connected to the trial) will make contact and parents will be informed of the trial, given the Parent information sheet (Appendix F) for the trial, and told about the possibility of the assessor. The information consultation will be held in an undisturbed setting, preferably the department meeting facilities. The parents will be given 24 hours to ask questions and make a decision, before a written informed consent can be obtained from both parents. Parents will be given a copy of the informed consent. If the birth is sudden the time to make a decision will be shorter.

9.2 Risk of complication for participants

The following procedure will be implemented to prevent and/or minimise risk of complication for participants.

Related to devices

Correct reading of the monitor (mainly to prevent false reading which may lead to wrong treatment) is critical. The investigators will train local staff as appropriate and trained staff will closely supervise all participants during the intervention.

To minimise skin irritation related to the device, the oximeter will be moved to different location every 4 hours.

SafeBoosC phase II

Related to application of the treatment guideline

All investigators have been involved in the development of the guideline and have approved the final version. The treatment guideline does not contradict any current national clinical practices. The investigators will train local staff as appropriate and will be available for consultation as needed.

9.3 Benefit for participants

The participants in both groups will receive careful attention from qualified physicians and hospital staff during the trial and a closer follow-up after hospital discharge compared to infants not enrolled into the trial.

10. Statistical plan and data analysis

10.1 Sample size estimation

An unpublished dataset of cerebral NIRS oximetry in 23 extremely preterm infants monitored from the first hours after birth until 72 hours after birth by the INVOS 4100 NIRS device in Utrecht during the period January 2004 until January 2008 was used to estimate the sample size (unpublished data). The cerebral saturation was recorded in 5 second-values. The data were censored by the proprietary Signal Base software. The recording was reliable 79% of the time, ranging from 48% to 97%. The time spent with a cerebral saturation below 55% and above 85% was calculated and the burden of hypo- and hyperoxia was calculated by summing the percentage deviation over time area under the curve – AUC). The burden of hypo- and hyperoxia was corrected for the loss of recording time and expressed as %hours per 72 hours.

There was a relatively high correlation between time spent in hypoxia and the burden of hypoxia. Overall, hyperoxia was only about 10% of hypoxia. There was a trend towards inverse correlation between hypoxia and hyperoxia. The distribution of AUC outside the range was skewed towards the right (higher values). The mean was 76.0%hours ± SD of 83.2. After a log-transformation, the distribution was not significantly different from normal. The mean was 1.64 ± SD of 0.50 (Figure 4).

SafeBoosC phase II

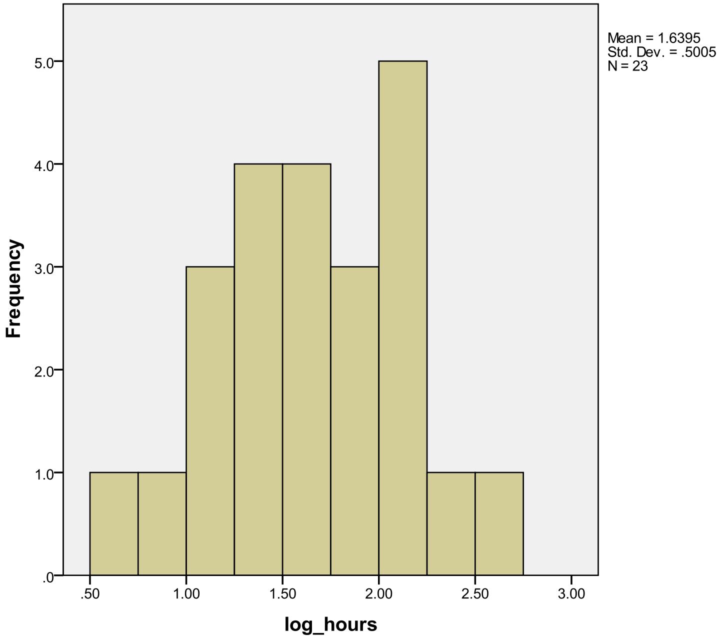

phase II

Figure 4: The distribution of the primary outcome burden of hypo-and hyperoxia expressed as %hours and transformed logarithmically

As a result, the statistical distribution has to be normalised by logarithmic transformation. Reducing the duration of time with hypoxia or hyperoxia by 0.30 (corresponding to a 50% reduction of the time with hypoxia or hyperoxia) with a type I error (alpha) of 5% and a type II error of 0.05 (power of 95%) requires randomisation of a total of 150 preterm infants (or ‘pair’ or ‘groups of infants’ in case of multiple births): 75 preterm infants in the experimental group and 75 preterm infants in the control group.

However the prevalence of twins among extremely preterm infants is about 30%. The within cluster correlation diminishes the statistical power. To account for this the sample size needs to be multiplied by the ‘design effect’ (50):

Design effect = 1 + (n’ – 1) x ICC

ICC = intraclass correlation coefficient

n' = average cluster size

The twin ICC is unknown for cerebral oximetry. A pragmatic solution is to increase the sample size to 166 in present study with a continuous outcome and only about 30% of infants in clusters of two. This corresponds to a ICC of 0.33.

10.2 Data analysis and statistical methods

All analyses will be intention to treat analyses conducted blinded with two sided tests at the 0.05 level of significance.

The statistical analysis is complicated by the fact that up to 30% of the births may be twin or triplet births where participating siblings will be randomized to the same arm creating multiple clusters of two observations that may be correlated. Shaffer et al (51) found that for this type of data without covariates added a mixed effects model with random intercept is the preferred model for continuous outcome data while ordinary logistic regression is preferred for binary data. Sauzet et all (52) studied the effect of including adjusting covariates in the model for a continuous outcome variable. They found not negligible and similar bias for estimated coefficients whether a mixed model or a linear regression model was used. Standard errors were underestimated using linear models thus inflating the type I error.

SafeBoosC phase II

A mixed model with random intercept for continuous outcome variables and ordinary logistic regression for binary (and ordinal) outcome variables will be used. The analyses will be adjusted for the protocol specified variables (gestational age category and trial site indicator) and (following multiple imputations if necessary) will be the primary results. Since the intra cluster correlation may cause parameter estimate bias in the presence of covariates

(other that the intervention indicator) for continuous outcome variables an unadjusted analysis will also be done. If the results of the adjusted and unadjusted analysis differ markedly the results will be discussed. The effect of covariates is unknown for binary outcome variables.

If for a specified statistical model to be used in one of the above analyses of primary and secondary outcome measures Little's test is significant ($p < 0.05$) and the percent missing cases > 5% multiple imputations will be applied to adjust for values missing at random (SPSS version 17 or later).

To assess the spectrum of potential bias resulting from data missing not at random the following sensitivity analysis will be done for the primary outcome measure: let A be the group with a beneficial effect (low outcome value) as compared to the other group (group B), min be the minimum value, and max the maximum value in the material. Two estimates of the coefficient (c) of the intervention indicator will then be calculated where missing values in A are replaced by the maximum value found in the material and missing values in B are replaced by minimum value found in the material and vice versa. Since the imputation may impact the standard error of the parameter estimate in an unpredictable way the standard error of the primary analysis will be used in each case to test if the estimate deviates significantly from 0.

SafeBoosC phase II

The null hypothesis corresponding to the primary outcome measure is tested at the 0.05 level of significance. If the test is significant the three secondary outcomes (IBI at 64 hours, cUS, all cause mortality at term) will be tested using alfa = 0.05 and Hommel's procedure as adjustment for multiplicity (53).

11. Data management

11.1 Data handling and archiving

All participant data are protected in accordance with the Danish Act on processing of personal data and the Danish Health Act. Source data will be registered in the participant's medical records or directly into the eCRF. A common web-based electronic case report form (eCRF) will be devised to enable a central database. Data entry into the central database handling of medical records is the responsibility of the investigators. Data will be stored in accordance with guidelines issued by the Danish Data Protection Agency, with which the trial will also be registered. After the establishment of a 'clean file', the database will be locked, data will be send for statistical analysis at the Copenhagen Trial Unit. The trial database will hereafter be kept according to the respective national laws. After end of trial, the data will be archived for five years according to good clinical practice guideline (appendix I).

At each trial-site the data flow (figure 5) will be monitored according to the GCP principles by a locally appointed external monitoring committee.

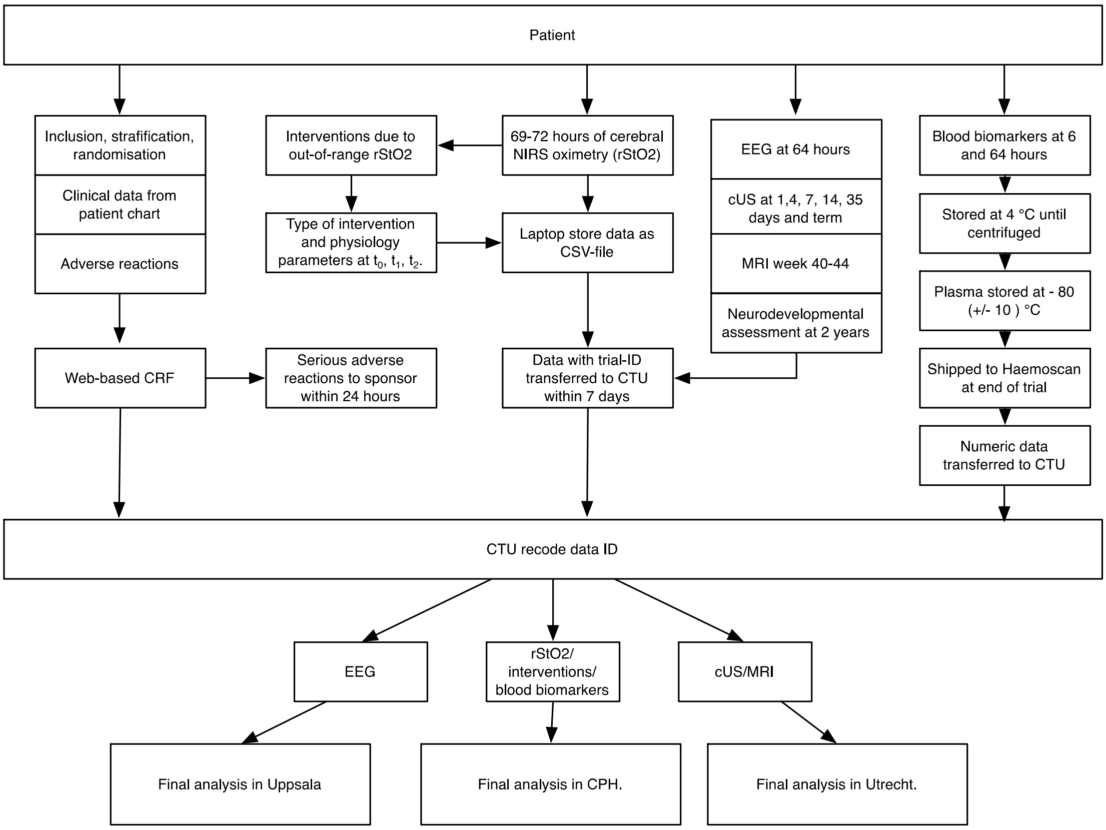

Figure 5: The data flow in SafeBoosC

After completion of statistical data analysis, data will be pseudo-anonymised and deposited at the Danish Data Archive. Also, data will be made available for other researchers by uploading a completely anonymised dataset onto ClinicalTrials.gov.

The investigator(s) permits trial-related monitoring, audits, regulatory inspection(s) by providing direct access to the source data and other relevant documents. Trial data will be handled according to regulations of data protection agency in the respective countries.

11.2 Medical coding

Adverse reactions (ARs) will be coded using MedDRA coding system by lowest level term (LLT), preterm term (PT), and system organ class (SOC). Concomitant medications will not be coded.

12. Quality assurance

The trial will be carried out in accordance with the Declaration of Helsinki in its latest form and the ICH GCP guidelines as well as national laws and regulations.

12.1 Monitoring

The trial will be monitored according to the ICH GCP guidelines, and a detailed monitoring plan will be developed. Centrally eCRFs will be checked, and as a minimum the following will be monitored locally:

- All patients for existence
- All patients for documented informed consent.

SafeBoosC phase II

12.2 Device quality control

Each site is responsible for adhering to the quality control measures described in the oximeter manufacturer's users guidelines.

13. Trial and funding timeframe

Trial stages	Timeframe
CIP development	Spring 2011
CIP finalised (first draft)	May 31, 2011
Site selection	Ongoing – depend on finance
Recruitment phase	May 2012 – May 2014
Assessment phase	Last participant recruited (Primary outcome May 2014)
Final analysis	2015

Trial stages	Timeframe
Publication	Late 2014 on primary and secondary outcomes

Funding	Timeframe
START	December 2010, awarded 183,848 DKK
Danish Strategic Research Council	November 2011, awarded 11,100.000 DKK

14. Legal aspects

14.1 Finance

The SafeBoosC project has received 183,848.00 DKK from the Danish Council for Strategic Research (DSF) under the Preparation of International Application (START) theme in December 2010. An application to the DSF seeking for 10,320,640.00 DKK under the Strategic Research in Individuals, Disease and Society theme, and was awarded 11,100,000.00 DKK November 2011. The sponsor/principal investigator Gorm Greisen is the initiator of the SafeBoosC project. He has no financial interest in the results of the trial, nor the NIRS-instruments. He is not financially or in any other way involved in the Danish Council for Strategic Research.

SafeBoosC phase II

14.2 Participant insurance

The participants will be insured in accordance with existing legislation of their respective country.

14.3 Publication plan

The trial will be registered on ClinicalTrials.gov prior to the randomisation of the first participant. Attempts will be sought to publish all results, positive, neutral, as well as negative, in a peer-reviewed international journals. Authorship will be determined according to the International Committee of Medical Journal Editors. Attempts will be made to publish a list of all investigators with their contributions in all publications.

14.4 Statements of compliance

The clinical investigation will be conducted in accordance with the ethical principles that have their origin in the Declaration of Helsinki.

The clinical investigation will comply with ISO14155 and the relevant national regulations of each participating medical centre, and will not begin until required approvals from ECs and regulatory authorities have been obtained. Additional requirements imposed by the ECs or regulatory authorities will be followed.

The clinical investigation will be conducted in accordance with this CIP.

Gorm Greisen

SafeBoosC phase II

15. Appendices

15.1 Appendix A: Treatment guidelines and justifications

SafeBoosC Clinical Guidelines

Assessment of cerebral oxygen saturation

Regional cerebral tissue oxygen saturation (rStO2) is a composite measure of tissue oxygen saturation across arterial, capillary and venous beds and reflects a balance between cerebral oxygen delivery (CDO2) and cerebral metabolic rate (CMRO2). In preterm infants, the CMRO2 is unlikely to vary much and a change in rStO2 largely reflects changes in CDO2. The factors which influence CDO2 are arterial oxygen saturation (SaO2), haemoglobin concentration ([Hb]), and cerebral blood flow (CBF).

Establishment of monitoring of cerebral oxygenation

As soon as possible and within 3 hours of age

Period of monitoring of cerebral oxygenation

Until 72 hours after birth

Instruction

Document the intervention chosen and any changes in rStO2 following intervention.

Recommendation for clinical interventions

The rStO2 target normal range is 55% to 85%. If the rStO2 is out of normal range and there is no reason to believe that it will normalise without intervention, consider one of the interventions (identified in '•') listed below and reassess 30 to 60 minutes after the intervention. Generally, only one intervention should be chosen at a time. All the interventions proposed here are commonly used in this patient group. After each intervention, the level of evidence for each intervention (I-III) and recommendation (A-E) are given (defined in table 1 and 2). For further explanation, see below.

SafeBoosC phase II

rStO2 < 55%

Aim of intervention: A low rStO2 reflects a low CDO2. The interventions should be directed to increasing SaO2, [Hb], and/or CBF.

<u>Assess cardiovascular status:</u>

Blood pressure below the normal range or low, even in the normal range, consider:

- Vasopressor-inotropes (I/B) (54,55)
- Fluid bolus (normal saline) (I/C) (56,57)
- Decrease mean airway pressure (III/B) (58-61)

Poor systemic circulation, consider if:

Echocardiography shows low cardiac output and/or low SVC flow

- Inotropes (I/B) (57,62-66)
- Fluid bolus (normal saline) (I/C) (56,57)
- Decrease mean airway pressure (III/B) (58-61)
- Reduce vasopressor (III/ B) (67)

Echocardiography not available but has at least 2 of the following signs:

Lactate > 3.5 mmol/l
CRT > 3 seconds
Urine output < 1 ml/kg/hour

consider:

- Inotropes (I/B) (57,62-66)
- Fluid bolus (normal saline) (I/C) (56,57)
- Decrease mean airway pressure (III/B)) (58-61)
- Reduce vasopressor (III/B) (67)

Patent ductus arteriosus, consider:
- Medical treatment (II-2/B) (44,59,60,68)

Assess oxygen transport:
Haemoglobin below the normal range or low, even in the normal range, consider:
Red blood cell transfusion (I/B) (69-72)

Assess respiratory status:
SaO2 below the normal range or low, even in normal range, consider:
- Increase FiO2 (II-1/A) (73) (ATTENTION: be careful not to exceed the upper target threshold of SpO2)
- Increase mean airway pressure (III/B) (58,74,75)

PCO2 below the normal rangen or low, even in normal range, consider:
- Decrease minute ventilation (II/A) (69,76-78)

rStO2 > 85%
Aim of intervention: A high rStO2 reflects impaired oxygen utilisation and/or disturbed cerebral autoregulation (hyperaemia) and interventions should be directed at identifying and treating the underlying cause.

Assess respiratory status:
SaO2 above the normal range or high, even in normal range, consider:
Decrease FiO2 (II-2/A) (79-83)
Decrease mean airway pressure (III/B) (74,75)

SafeBoosC phase II

PCO2 above the normal range or high,, even in normal range, consider:
Increase minute ventilation (II/A) (69,76-78)

Assess blood glucose level:
Blood glucose < 2.5 mmol/l, consider to:
Increase glucose intake (II/A) (84,85)

Level of evidence and recommendation of intervention
The level of evidence (Table 1) and recommendation for a given intervention (in brackets and Table 2)) were graded according to the U.S. Preventive Services Task Force system (86).

Table 1: Hierarchy of research design and level of evidence

Level of evidence	Type of study
I	Evidence obtained from at least one properly randomized controlled trial
II-1	Evidence obtained from well-designed controlled trials without randomization
II-2	Evidence obtained from well-designed cohort or case-control analytic studies, preferably from more than one centre or research group
II-3	Evidence obtained from multiple time series with or without intervention. Dramatic results in uncontrolled experiments (such as the results of the introduction of penicillin treatment in the 1940s) could also be regarded as this type of evidence
III	Opinions of respected authorities, based on clinical experience,

	descriptive studies and case reports, or reports of expert committees

Table 2: Recommendation grid

	Net benefit			
Quality of evidence	substantial	moderate	small	zero/negative
Good	A	B	C	D
Fair	B	B	C	D
Poor	E	E	E	E
Standard recommendation language	A= Strongly recommended (good evidence that the intervention improves important health outcomes and benefits substantially outweigh harms).<br>B= Recommended (at least fair evidence that the intervention improves important health outcomes and benefits substantially outweigh harms).<br>C= No recommendation for or against routine provision of the intervention (fair evidence that the service can improve health outcomes but the balance of the benefits and harms is too close to justify a general recommendation).<br>D= Recommends against routinely providing the intervention (at least fair evidence that the service is ineffective or that harms outweigh benefits).<br>E= Insufficient to recommend for or against routinely providing the intervention (evidence that the intervention is effective is lacking, of poor quality, or conflicting and the balance of benefits and harms cannot be determined).			

SafeBoosC phase II

Abbreviations

SVC	*Superior vena cava*
CRT	*Capillary refill time*
FiO2	*Fraction of inspired oxygen*
rStO2	*Regional cerebral tissue oxygen saturation*
SaO2	*Saturation of Oxygen (arterial blood)*
PCO2	*Partial Pressure of Carbon Dioxide*
CDO2	*Cerebral oxygen delivery*
CMRO2	*Cerebral metabolic rate*
[Hb]	*Haemoglobin concentration*
CBF	*Cerebral blood flow*

15.2 Appendix B: Procedure for assessment of chemical biomarkers

OUTCOME MEASURES
Outcome measure 1: S100β
Outcome measure 2: BFABP
Outcome measure 3: Neuroketal

MEDIUM
Blood
If a central blood catheter is in place up to one ml of blood will be collected in **heparin**. Commercially available tubes containing lithium or sodium heparin are available to collect 2 ml of blood. These tubes can be used without vacuum and filled with 1 ml from a syringe. The higher final heparin concentration is no problem. Tubes with a gel to aid separation of plasma may be used, and must be used if less than 1 ml is drawn (if using gel separator tubes, follow the manufacturers instructions leaflet) .
EDTA or citrate plasma cannot be used for measurement of S100β. Blood must be kept cold after collection on ice or in the fridge at 4ºC ±2ºC until it is centrifuged (1200xg for 12 minutes in a normal centrifuge or 13000 rpm in a microcentrifuge) to obtain platelet poor plasma. As much as possible plasma is then collected, transferred to small screw-cap tubes and stored frozen (-20ºC ±4ºC for a maximum of one week, thereafter at -80ºC ±10ºC for prolonged storage).

Urine
If possible, 1 ml of urine shall be collected and stored frozen (-20ºC ±4ºC for a maximum of one week, thereafter at -80ºC ±10ºC for prolonged storage) without further treatment.

TIME OF COLLECTION
Blood: (can be +/-1 hours of specified time points):
- 6 hours after birth, and
- 64 hours after birth

Urine (if possible) - (can be +/-1 hours of specified time points):
- 6 hours after birth, and
- 64 hours after birth.

SafeBoosC phase II

STORING
Samples should be labeled with two country letters, identification (randomisation number), Plasma or Urine, time points .. Example: DK 5 P 2 = Rigshospitalet participant 5, plasma collected after 64 hrs. The principal investigator shall keep record of the participant identity linked to the participant number. All samples should be stored at the site until last participants samples has been collected and recorded.

SHIPPING:
When last participant has been enrolled, samples should, immediately hereafter, be shipped in batches on dry ice, using DHL as a courier. Shipping will always be Monday or Tuesday.
To the following address:

Haemoscan B.V.
Stavangerweg 23, Groningen
9723 JC, The Netherlands
Att: Wim van Oeveren

Phone : +31(0)646181604
Fax nr +31 (0)847269747

Material transfer agreements between Haemoscan and the sites will be issued by each institutions legal department.

METHODS OF ASSESSMENT
All assessments are conducted without knowledge of allocation group (experimental or control).

S100β
The S100β serum-concentration will be assesed by ELISA. It can be detected in heparin plasma, heparin serum or urine in 50 µl samples.

BFABP
Brain fatty acid binding protein (BFABP) is determined by means of ELISA with BFABP specific monoclonal capture antibodies and polyclonal detection antibody in plasma/serum as well as urine. 100 µl is needed for a BFABP ELISA, due to its low concentrations.

Neuroketal
Determination of neuroketal is performed by competitive enzyme immunoassay in 100 µl of plasma/serum or urine.

Abbreviations

S100β	Acidic calcium binding protein found in the nervous system
BFABP	Brain fatty acid binding protein
ELISA	Enzyme linked immunosorbent assay
ml	Millilitre

SafeBoosC phase II

15.3 Appendix C: Procedures for assessment of aEEG/EEG

OUTCOME MEASURES

Outcome measure 1: Interburst interval (IBI)
Outcome measure 2: Power in delta band
Outcome measure 3: Power in theta band
Outcome measure 4: Power in alpha band
Outcome measure 5: Power in beta band
Outcome measure 6: Background pattern
Outcome measure 7: Sleep wake cycling
Outcome measure 8: Seizures

MEDIUM
Raw EEG-data

TIME OF COLLECTION
Single-channel EEG is applied for at least 120 minutes at 56-72 hours after birth. This should be at least 3 hours after administration of bolus morphine, if possible. Hydrogel electrodes or needle electrodes may be used. P3 and P4 positions according to the international 10–20 system; a frontal reference electrode is also applied.

METHOD OF ASSESSMENT OR SCORING
The EEG is divided in 10-minutes epochs. The quality of all EEG epochs is visually assessed without knowledge of allocation group (experimental or control). Epochs showing artifacts that could affect the quantitative analysis are discarded.

SafeBoosC phase II

IBIs are measured by using an automated algorithm and averaged in each 10-minute epoch. The IBI detector algorithm is based on a non-linear energy operator that reflects both amplitude and frequency content of the EEG. Power spectral analysis is performed using fast Fourier transformation with a time base of 10 seconds. For every epoch, total and relative (%) band power is calculated within the following frequency bands: delta (0.5–4 Hz), theta (4–8 Hz), alpha (8 –13 Hz), and beta (13–30 Hz).

If more than one 10 minutes epoch is analysed, the simple average of all measures is used for further analysis

Abbreviations

aEEG	Amplitude-integrated electroencephalogram
EEG	Electroencephalogram
IBI	Interburst interval
Hz	Hertz

15.4 Appendix D: Procedures for MRI examination

OUTCOME MEASURES
Outcome measure 1: Brain injury score according to Woodward with cerebellum score
Outcome measture 2: Volumetric measurements:
Outcome measure 3: Cortical folding with formation of the sulci:
Outcome measure 4: Diffusion tensor imaging:

MEDIUM
Cerebral MRI scan

TIME OF COLLECTION
At 40-44 weeks of gestational age.

METHOD OF ASSESSMENT of brain injury scoring and quantitative measurements
The MRI protocol consists of anatomical scans preferable on 3Tesla MRI (T1 3D weighted image and T2 weighted image), DWI and DTI. The T1 and T2 weighted images are performed in coronal slices and the T1-3D weighted image at around 100-110 slices. The DTI images are performed in axial slices covering the whole brain. The focus is on the following features: brain tissue volumes, including myelinisation, cortical folding with formation of the sulci, in relation to DWI/DTI. All assessments are conducted without knowledge of allocation group (experimental or control).

Brain injury score according to Woodward plus cerebellum:
The scoring system for the composite white matter and gray matter scores consists of eight assessments each using a 3-point scale.
White matter abnormality is graded using five areas of assessment within the white matter with a score of 1 (normal), 2 (mild abnormality) and 3 (moderate-severe abnormality) for each area of assessment. The five areas of white matter abnormality that are scored include:

1. ***The nature and extent of white matter signal abnormality -*** This is scored based on the T1- and T2-weighted signal abnormalities in the white matter best observed in the axial imaging.
Grade 1: normal T1- and T2-weighted signal throughout the white matter.
Grade 2: focal regions of high T1-or T2-weighted signal (2 or fewer regions per hemisphere).
Grade 3: multiple regions of high T1- or T2-weighted signal (more than 2 regions per hemisphere).

2. ***Periventricular white matter volume loss*** – This is also scored based on the combined T1- and T2-weighted imaging analyzing the ratio of periventricular white matter to ventricular volume in both the axial and sagittal imaging.
Grade 1: normal periventricular white matter volume usually associated with small ventricles
Grade 2: mild reduction in white matter volume with mild to moderate increased ventricular size.
Grade 3: marked reduction in white matter volume often occurring with marked increase in the size of the ventricle and/or extra-axial space.

3. ***Cystic abnormalities*** – This is easily assessed on any imaging view or modality but occurs rarely.
Grade 1: normal, with no cystic abnormality.
Grade 2: less than 2mm single focal cyst.
Grade 3: multiple cysts or a single larger (2mm or greater) single focal cyst is rare.

4. ***Ventricular dilatation*** – This is also scored based on the combined T1- and T2-weighted imaging in both the axial and sagittal imaging.
Grade 1: normal with no evidence of ventricular dilatation.
Grade 2: moderate enlargement resulting in mild rounding of the frontal horns, minimal enlargement of the temporal horns and moderate enlargement of the occipital horns.
Grade 3: more global enlargement of a moderate to severe nature including significant enlargement of the frontal, temporal and occipital horns.

SafeBoosC phase II

5. ***Thinning of the corpus callosum –*** This is scored based on the sagittal and coronal T1-weighted MR imaging.
Grade 1: normal, with thick corpus callosum visible in all views.
Grade 2 :focal thinning in the corpus callosum often visible in the mid region of the body of the corpus on sagittal images.
Grade 3 :global thinning across the entire corpus callosum.

5 assessments of the cerebral white matter are then combined to give an overall white abnormality score which is categorized as:

- no abnormality - Total score 5 to 6
- mild abnormality – Total score 7 to 9
- moderate abnormality – Total score 10 to 12
- severe abnormality – Total score 13 to 15.

Gray matter abnormality is graded using three scales assessing:
1) ***Presence of gray matter cortical signal abnormality*** . This is very rarely detected and thus does not contribute significantly to the overall scoring. It is scored on high signal intensity in the cortex on axial T1 and/or loss of cortical ribbon signal on axial T2-weighted MR imaging.
2) ***Quality of gyral maturation*** – Cortical gyration maturation is rated by standard gyral model (3,4) using representative MRI images and cerebral models of gyral development at 34 weeks, 36 weeks, 38 weeks and 40-42 weeks on axial T1- and T2-weighted MR imaging. The gyral pattern assessment in these maturational assignments includes at 34-36 weeks the presence of marginal sulcus, paracentral gyrus with secondary sulci present in the frontal lobes, superior and middle temporal, rolandic and occipital regions. By 36-38 weeks the gyral pattern should include additional secondary gyri in the transverse and inferior temporal and cingulated gyri. At 40 weeks' gestation there would be tertiary inferior temporal and inferior occipital gyri and sulci
Grade 1: normal for 40 weeks.
Grade 2: 2 to 4 weeks delay in gyral development (i.e. consistent with 36 to 40 weeks).
Grade 3: more than 4 weeks delay in gyral development.
3) ***Size of the subarachnoid space -*** This is scored based on the sagittal T1 and coronal T1- or T2-weighted MR images for the size of the subarachnoid space in relation to the cerebral mantle.
Grade 1: small subarachnoid space which is barely visible.
Grade 2:mildly enlarged CSF space with visible enlargement of the space between the major sulci and the interhemispheric space in addition to the extracerebral space.
Grade 3: more substantially enlarged global subarachnoid space with visible cerebrospinal fuid between many gyri as well as interhemispheric and extracerebral.

SafeBoosC phase II

The total gray matter score was then calculated and categorized as
(a) normal - score 3 to 5
(b) abnormal score 6 to 9.

Cerebellar lesions:

(a) normal: no lesions
(b) mild: < 6 punctate lesions
(c) moderate /severe: > 6 punctate lesions/cerebellar hemorrhage

Volumetric measurements: The segmentation is performed on the MR images of individual participants. The segmented tissue types include: unmyelinated and myelinated white matter, cortical and central gray matter (basal ganglia and thalamus), cerebro-spinal fluid around the brain, ventricles, cerebellum and brainstem. This segmentation method was developed for neonates (87). Quantitative measurements of the cerebral tissue volumes are performed by the knn-approach segmentation method which is based on the probability of each tissue classes from signal intensities in T1- and T2-weighted images and the anatomic location. Thereafter, the volume of each tissue is quantified.

Cortical folding with formation of the sulci: For cortical folding, the inner cortical surface is segmented and reconstructed in 3D from the T2-weighted images. The surface is detected between the developing cortex and white matter zone. The global area of this inner cortical surface is then computed. The segmentation of the inner surface is used as it highlights the cortical sulcation pattern more precisely than the outer surface, particularly where sulci are still not deeply folded. Finally, the local surface curvature is estimated from the mesh local geometry: positive curvatures correspond to the gyri top, and negative curvatures to the folds bottom. So the 3D image is then created. The sulci are defined as connected components of negative curvature and labelled manually according to post-mortem and MRI atlases and prenatal images. To characterize the sulci maturation according to gestational age, their sulci areas will be calculated. Thereafter, for each infant, the sulcation index with total cortical surface and thickness can be computed. Sulcation index is defined as the ratio between the areas of sulci and the surface of the total cortex. This surface increases with the age-related brain growth. The sulcation index, thus, characterizes the proportion of sulci according to the whole brain size, and is a measure of maturation of the cortex.

Diffusion tensor imaging: (DTI) scans are obtained to perform analyses of the diffusion coefficients on the brain averages, as well as on individual participants. It visualizes the movement of fluid in the brain, and can be used to identify white matter tracts. We will study the maturation of white matter fiber tracks in relation to cortical development.

Abbreviations

DWI	Diffusion weighted imaging
DTI	Diffusion tensor imaging
MRI	Magnetic resonance imaging
T1	T1-weighted
T2	T2-weighted
3D	Three-dimensional

SafeBoosC phase II

15.5 Appendix E: Procedure for assessment of cranial ultrasound

OUTCOME MEASURES
Outcome measure 1: Parenchymal/periventricular haemorrhagic infarction / inhomogeneous flaring
Outcome measure 2: IVH Grade III
Outcome measure 3: Cerebellar haemorrhage
Outcome measure 4: Posthaemorrhagic hydrocephalus
Outcome measure 5: cPVL
Outcome measure 6: Cerebral atrophy

MEDIUM
Sonogram

Coronal:
C1 frontal lobes
C2 foramen Monro
C3 lateral ventricles with largest size of VI
C4 cerebellum largest diameter left-right
C5 posterior horns with plexus
C6 periventricular

Sagital
S1 midline
S2 left lateral ventricle
S3 left periventricular through fissura Sylvi
S4 right lateral ventricle
S5 right periventricular throuh fissura Sylvi

SafeBoosC phase II

Cerebellum via mastoid with tranducer in coronal position such as coronal view via anterior fontanel

TIME OF COLLECTION
- Early scans (day 1,4):
- Late scans (day 7, 14, 35)
- Term scan: 40-44 weeks of gestational age

METHOD OF ASSESSMENT OR SCORING
The assessment classification is:
1. Normal brain scan
- No cysts
- No ventricular dilatation
- No enlargement of extra-cerebral spaces
- Normal cortical grey matter

2. Mild brain injury:
- Grade 1-2 IVH (including GLH)
- Persistent pathologic non-decreasing inhomogeneous flaring at day 7 and 14
- Thinning of the corpus callosum
- Ventriculomegaly at term, ventricular index < p97

3. Severe brain injury will be defined as
- Intraventricular haemorrhage grade III (ventricular index > p97 during the acute phase)
- Posthaemorrhagic ventricular dilatation

- Parenchymal/periventricular haemorrhagic infarction
- Local cystic lesions (unilateral)
- Cystic periventricular leukomalacia (bilateral)]
- Cerebellar haemorrhage
- Cerebral atrophy at term age (ventricular dilation and/or increased extra-cerebral spaces

All assessments are conducted without knowledge of allocation group (experimental or control).

Abbreviations

IVH	Intraventricular haemorrhage
cPVL	Cystic periventricular leucomalacia
cUS	Cranial ultrasound

SafeBoosC phase II

15.6 Appendix F: Parental Information

For Danish version please separate document:
SafeBoosC_deltagerinfomation_ dansk_Appendix F (vs.1.1)

15.7 Appendix G: Informed consent – the SafeBoosC phase II trial

For Danish version please separate document:
SafeboosC_ samtykke_dansk_ Appendix G (vs.1.1)

15.8 Appendix H: Parental information - the SafeBoosC trial -MRI

For Danish version please separate document:
SafeBoosC_deltagerinformaton_ MRI_ dansk_Appendix H (vs.1.1)

15.9 Appendix I: Informed consent- the SafeBoosC trial -MRI

For Danish version please separate document:
SafeBoosC _samtykke_ MRI_dansk_Appendix I (vs.1.1)

SafeBoosC phase II

15.10 Appendix J: SafeBoosC - Data flow

SafeBoosC - Data flow

Web-based Case Report Form

The CRF will be a web-based solution in the open source clinical trials software OpenClinica®. This will handle the inclusion procedure, the documentation of the stratification and randomisation process, the adverse reaction and the relevant clinical data from the patient apart from $rStO_2$ and interventions plus physiological parameters. The data will be entered into the CRF by keying by the medical staff. A form for the inclusion, the randomisation, the NIRS monitoring period, the term follow up, and the two-year follow up will be created. Serious adverse reactions will be reported directly to the sponsor within 24 hours.

Regional tissue oxygen saturation $rStO_2$

Each oximeter will be connected to a laptop computer through serial port that will display or blind the values of $rStO_2$. A dedicated software solution designed to SafeBoosC data acquisition will handle the NIRS data. The 0.2 Hz $rStO_2$ numeric values will be stored continuously in CSV file with a data and time code on the laptop throughout the monitoring period of 72 hours. If the oximeter provides information on signal quality this will be recorded simultaneously. The CSV file will be labelled with the patient trial identification number (trial-ID). At the end of the monitoring period the CSV file of 5-10 MB will be uploaded to Copenhagen Trial Unit (CTU) that will store the data and perform the data analysis. If the file is not uploaded within 2 workdays the relevant investigator will be contacted.

Treatment guideline interventions

If interventions from the treatment guideline are initiated due to an out-of-range $rStO_2$ the type of intervention, the current values of $rStO_2$, SpO_2, MABP, and pCO_2 will by entered by the

attending medical staff by simple keying into a software solution designed to SafeBoosC data acquisition. Depending on the intervention the data acquisition software will prompt the staff to enter the values again 30 and 60 minutes or 60 and 240 minutes afterwards. Each possible intervention will be given specific number. The interventions and values will be recorded and stored in the same CSV file as the $rStO_2$ values and uploaded to Copenhagen Trial Unit at the end of monitoring period.

Cerebral Ultrasound
Cerebral ultrasound will be done at 1 and4 days after birth, and again at 7, 14, and 35 days, and finally at term date. 11 pre-specified views are to be recorded and stored. After each cerebral ultrasound examination the digital pictures 20 - 100 MB will be labelled with the trial-ID and uploaded to the CTU for storage. If the pictures are not uploaded within one month after the last cUS examination or are insufficient the relevant investigator will be contacted. The files' ID will be recoded before transfer to Wilhelmina Children's Hospital in Utrecht, Holland, for centralised final analysis.

Blood biomarkers
Up to one ml of blood will be collected in heparin at time points 6 hours ±1H after birth and 64 hours±1H after birth. Blood will be kept cold after collection on ice or in the fridge at 4ºC ±2ºC until it is centrifuged (1200xg for 12 minutes in a normal centrifuge or 13000 rpm in a micro-centrifuge) to obtain platelet poor plasma. As much as possible plasma is then collected, transferred to small screw-cap tubes, and stored frozen (-20ºC ±4ºC for a maximum of one week, thereafter at -80ºC ±10ºC for prolonged storage).

Samples will be labelled with trial ID, and time points. All samples will be stored at the site until last participants samples has been collected and recorded. When last participant has been enrolled, samples will be shipped in batches on dry ice, using DHL as a courier to the Haemoscan B.V., Groningen, Holland. The numeric results of the biomarkers concentration in blood will be transferred to Rigshospitalet, Copenhagen, Denmark, for final, centralised analysis.

SafeBoosC phase II

Electroencephalogram
Single-channel EEG is applied for at least 120 minutes at 56-72 hours after birth. Electrodes are placed at positions P3 and P4 according to the international 10–20 system; a frontal reference electrode is also applied. The raw data file (5-10 MB) is labelled with the trial-ID and up-loaded to the CTU. The ID of the files will be recoded before transfer to Dept. Of Neonatology, Rigshospitalet, Copenhagen, Denmark for centralised final analysis.

Magnetic resonance imaging
Magnetic resonance imaging (MRI) will be done at term age. The raw data (100-400 MB) will be stored locally on a cd-rom. Data labelled with trial ID will also be uploaded to CTU within one month after conducting the MRI. CTU will recode the ID before shipment to Wilhelmina Children's Hospital in Utrecht, Holland, for centralised final analysis.

Flow chart of SafeBoosC data flow:

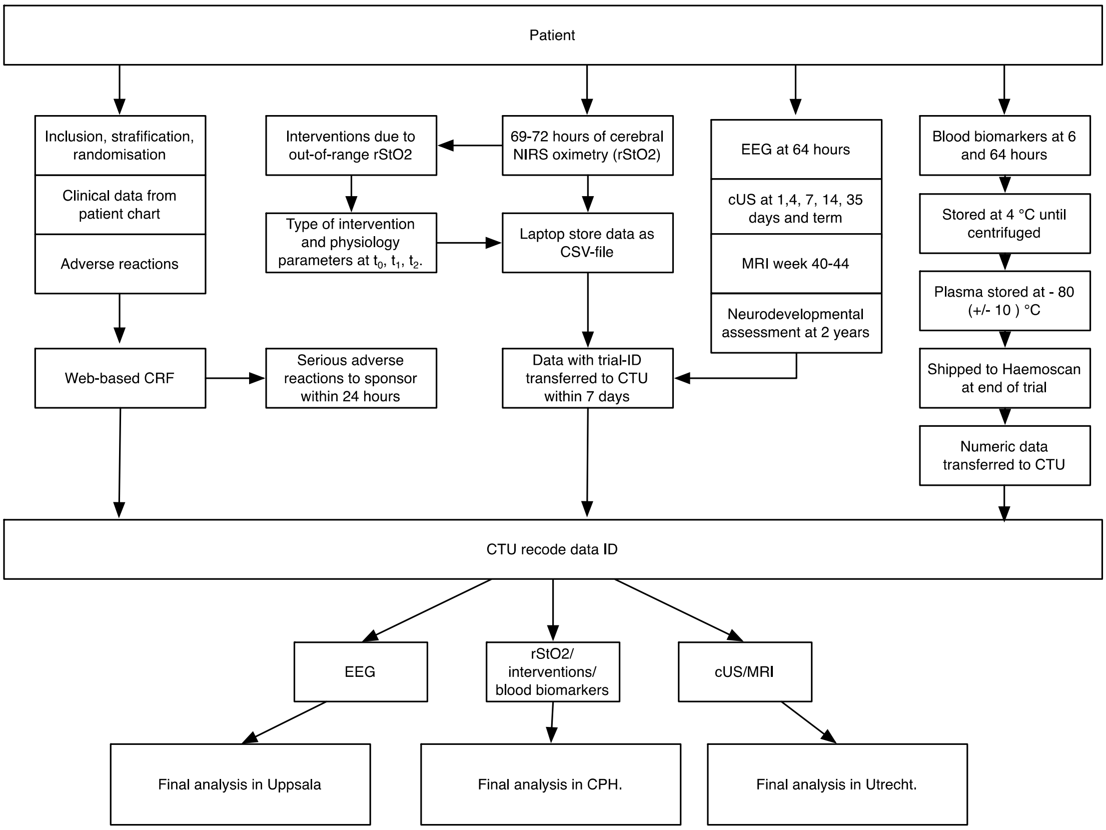

SafeBoosC phase II

16. References

1. Volpe JJ. Brain Injury in the Premature Infant: Neuropathology, Clinical Aspects, and Pathogenesis. Ment Retard Dev Disabil Res Rev. 1997 Jun 5;(3):3–12.

2. Kluckow M. Low systemic blood flow and pathophysiology of the preterm transitional circulation. Early Hum Dev. 2005 May 1;81(5):429–37.

3. Cayabyab R, Mclean CW, Seri I. Definition of hypotension and assessment of hemodynamics in the preterm neonate. Journal of perinatology : official journal of the California Perinatal Association. 2009 May 1;29 Suppl 2:S58–62.

4. Guzzetta F, Shackelford GD, Volpe S, Perlman JM, Volpe JJ. Periventricular intraparenchymal echodensities in the premature newborn: critical determinant of neurologic outcome. Pediatrics. 1986 Dec;78(6):995–1006.

5. Volpe JJ. Brain injury in premature infants: a complex amalgam of destructive and

developmental disturbances. Lancet Neurol. 2009 Jan 1;8(1):110–24.

6. Perlman JM. White matter injury in the preterm infant: an important determination of abnormal neurodevelopment outcome. Early Hum Dev. 1998 Dec;53(2):99–120.

7. Greisen G, Vannucci RC. Is periventricular leucomalacia a result of hypoxic-ischaemic injury? Hypocapnia and the preterm brain. Biol Neonate. 2001;79(3-4):194–200.

8. Miall-Allen VM, de Vries LS, Whitelaw AG. Mean arterial blood pressure and neonatal cerebral lesions. Arch Dis Child. 1987 Oct 1;62(10):1068–9.

9. Perlman JM, Volpe JJ. Are venous circulatory abnormalities important in the pathogenesis of hemorrhagic and/or ischemic cerebral injury? Pediatrics. 1987 Nov;80(5):705–11.

10. van Bel F, Ouden den L, van de Bor M, Stijnen T, Baan J, Ruys JH. Cerebral blood-flow velocity during the first week of life of preterm infants and neurodevelopment at two years. Dev Med Child Neurol. 1989 Jun;31(3):320–8.

11. Toet MC, Lemmers PMA, van Schelven LJ, van Bel F. Cerebral oxygenation and electrical activity after birth asphyxia: their relation to outcome. Pediatrics. 2006 Feb;117(2):333–9.

12. Dempsey EM, Barrington KJ. Treating hypotension in the preterm infant: when and with what: a critical and systematic review. Journal of perinatology : official journal of the California Perinatal Association. 2007 Aug 1;27(8):469–78.

SafeBoosC phase II

13. Horsch S, Muentjes C, Franz A, Roll C. Ultrasound diagnosis of brain atrophy is related to neurodevelopmental outcome in preterm infants. Acta Paediatr. 2005 Dec;94(12):1815–21.

14. Nongena P, Ederies A, Azzopardi DV, Edwards AD. Confidence in the prediction of neurodevelopmental outcome by cranial ultrasound and MRI in preterm infants. Arch Dis Child Fetal Neonatal Ed. 2010 Nov;95(6):F388–90.

15. de Vries LS, Van Haastert I-LC, Rademaker KJ, Koopman C, Groenendaal F. Ultrasound abnormalities preceding cerebral palsy in high-risk preterm infants. The Journal of Pediatrics. 2004 Jun;144(6):815–20.

16. O'Shea TM, Kuban KCK, Allred EN, Paneth N, Pagano M, Dammann O, et al. Neonatal cranial ultrasound lesions and developmental delays at 2 years of age among extremely low gestational age children. Pediatrics. 2008 Sep;122(3):e662–9.

17. Hellström-Westas L, Rosén I, Svenningsen NW. Predictive value of early continuous amplitude integrated EEG recordings on outcome after severe birth asphyxia in full term infants. Arch Dis Child Fetal Neonatal Ed. 1995 Jan;72(1):F34–8.

18. Toet MC, der Meij van W, de Vries LS, Uiterwaal CSPM, van Huffelen KC. Comparison between simultaneously recorded amplitude integrated electroencephalogram (cerebral function monitor) and standard electroencephalogram in neonates. Pediatrics. 2002 May;109(5):772–9.

19. Hellström-Westas L, Klette H, Thorngren-Jerneck K, Rosén I. Early prediction of outcome with aEEG in preterm infants with large intraventricular hemorrhages.

Neuropediatrics. 2001 Dec;32(6):319–24.

20. Wikstrom S, Lundin F, Ley D, Pupp IH, Fellman V, Rosén I, et al. Carbon Dioxide and Glucose Affect Electrocortical Background in Extremely Preterm Infants. Pediatrics. 2011 Apr 1;127(4):e1028–34.

21. Bayley N. Manual for the Bayley Scales of Infant Developmen. 1st ed. Psychological Corp; 1969.

22. Gollenberg AL, Lynch CD, Jackson LW, McGuinness BM, Msall ME. Concurrent validity of the parent-completed Ages and Stages Questionnaires, 2nd Ed. with the Bayley Scales of Infant Development II in a low-risk sample. Child Care Health Dev. 2010 Jul;36(4):485–90.

23. Menke J, Voss U, Möller G, Jorch G. Reproducibility of cerebral near infrared spectroscopy in neonates. Biol Neonate. 2003;83(1):6–11.

24. Wolf M, Greisen G. Advances in near-infrared spectroscopy to study the brain of the preterm and term neonate. Clin Perinatol. 2009 Dec 1;36(4):807–34, vi.

25. Brazy JE, Lewis DV, Mitnick MH, Jöbsis vander Vliet FF. Noninvasive monitoring of cerebral oxygenation in preterm infants: preliminary observations. Pediatrics. 1985 Feb;75(2):217–25.

26. Suzuki S, Takasaki S, Ozaki T, Kobayashi Y. A tissue oxygenation monitor using NIR spatially resolved spectroscopy. Proc SPIE. 1999;3597:582–92.

27. Watzman HM, Kurth CD, Montenegro LM, Rome J, Steven JM, Nicolson SC. Arterial and venous contributions to near-infrared cerebral oximetry. Anesthesiology. 2000 Oct 1;93(4):947–53.

28. Shah N, Trivedi NK, Clack SL, Shah M, Shah PP, Barker S. Impact of hypoxemia on the performance of cerebral oximeter in volunteer subjects. J Neurosurg Anesthesiol. 2000 Jul 1;12(3):201–9.

29. Kim MB, Ward DS, Cartwright CR, Kolano J, Chlebowski S, Henson LC. Estimation of jugular venous O2 saturation from cerebral oximetry or arterial O2 saturation during isocapnic hypoxia. J Clin Monit Comput. 2000;16(3):191–9.

30. Daubeney PE, Pilkington SN, Janke E, Charlton GA, Smith DC, Webber SA. Cerebral oxygenation measured by near-infrared spectroscopy: comparison with jugular bulb oximetry. ATS. 1996 Mar 1;61(3):930–4.

31. de Backer D, Ospina-Tascon G, Salgado D, Favory R, Creteur J, Vincent J-L. Monitoring the microcirculation in the critically ill patient: current methods and future approaches. Intensive Care Med. 2010 Nov;36(11):1813–25.

32. Dullenkopf A, Frey B, Baenziger O, Gerber A, Weiss M. Measurement of cerebral oxygenation state in anaesthetized children using the INVOS 5100 cerebral oximeter. Paediatr Anaesth. 2003 Jun;13(5):384–91.

33. Hyttel-Sørensen S, Sorensen L, Riera J, Greisen G. Tissue oximetry: a comparison of mean values of regional tissue saturation, reproducibility and dynamic range of four NIRS-instruments on the human forearm

.

SafeBoosC phase II

34. Yoshitani K, Kawaguchi M, Tatsumi K, Kitaguchi K, Furuya H. A comparison of the INVOS 4100 and the NIRO 300 near-infrared spectrophotometers. Anesth Analg. 2002 Mar 1;94(3):586–90; tableofcontents.

35. Thavasothy M, Broadhead M, Elwell C, Peters M, Smith M. A comparison of cerebral oxygenation as measured by the NIRO 300 and the INVOS 5100 Near-Infrared Spectrophotometers. Anaesthesia. 2002 Oct;57(10):999–1006.

36. Nagdyman N, Ewert P, Peters B, Miera O, Fleck T, Berger F. Comparison of different near-infrared spectroscopic cerebral oxygenation indices with central venous and jugular venous oxygenation saturation in children. Paediatr Anaesth. 2008 Feb;18(2):160–6.

37. Jenny C, Biallas M, Trajkovic I, Fauchere J-C, Bucher H-U, Wolf M. Reproducibility of cerebral tissue oxygen saturation measurements by near-infrared spectroscopy in newborn infants. J Biomed Opt. 2011;16(9):097004.

38. Mcneill S, Gatenby JC, Mcelroy S, Engelhardt B. Normal cerebral, renal and abdominal regional oxygen saturations using near-infrared spectroscopy in preterm infants. Journal of Perinatology. 2011 Jan;31(1):51–7.

39. Zhou CL, Liu Y-F, Zhang J-J, Xie L-J, Li Z-G, Wang D-H, et al. [Measurement of brain regional oxygen saturation in neonates in China: a multicenter randomized clinical trial]. Zhonghua Er Ke Za Zhi. 2009 Jul;47(7):517–22.

40. Taillefer M-C, Denault AY. Cerebral near-infrared spectroscopy in adult heart surgery: systematic review of its clinical efficacy. Can J Anaesth. 2005 Jan 1;52(1):79–87.

SafeBoosC phase II

41. Lemmers PMA, van Bel F. Left-to-right differences of regional cerebral oxygen saturation and oxygen extraction in preterm infants during the first days of life. Pediatr Res. 2009 Feb;65(2):226–30.

42. Lemmers PMA, Toet M, van Schelven LJ, van Bel F. Cerebral oxygenation and cerebral oxygen extraction in the preterm infant: the impact of respiratory distress syndrome. Exp Brain Res. 2006 Aug;173(3):458–67.

43. van den Berg E, Lemmers PMA, Toet MC, Klaessens JHG, van Bel F. Effect of the "InSurE" procedure on cerebral oxygenation and electrical brain activity of the preterm infant. Arch Dis Child Fetal Neonatal Ed. 2009 Dec 17;95(1):F53–8.

44. Lemmers PMA, Toet MC, van Bel F. Impact of patent ductus arteriosus and subsequent therapy with indomethacin on cerebral oxygenation in preterm infants. Pediatrics. 2008 Jan;121(1):142–7.

45. Lemmers PMA, Molenschot MC, Evens J, Toet MC, van Bel F. Is cerebral oxygen supply compromised in preterm infants undergoing surgical closure for patent ductus arteriosus? Arch Dis Child Fetal Neonatal Ed. 2010 Nov;95(6):F429–34.

46. Lemmers P. The clinical use of near infrared spectroscopy-monitored cerebral oxygen saturation and extraction in the preterm infant. Self-published thesis; 2010.

47. Wijbenga RG, Lemmers PMA, van Bel F. Cerebral oxygenation during the first days of life in preterm and term neonates: differences between different brain regions. Pediatr Res. 2011 Oct;70(4):389–94.

48. Pocivalnik M, Pichler G, Zotter H, Tax N, Muller W, Urlesberger B. Regional tissue

oxygen saturation: comparability and reproducibility of different devices. J Biomed Opt. 2011 May;16(5):057004.

49. E6 (R1) Guideline for Good Clinical Practice. The International Conference on Harmonisation [Internet]. 2009 Jul 13;:1–48. Available from: http://www.ema.europa.eu/docs/en_GB/document_library/Scientific_guideline/2009/09/WC500002874.pdf

50. Rao JN, Scott AJ. A simple method for the analysis of clustered binary data. Biometrics. 1992 Jun;48(2):577–85.

51. Shaffer ML, Kunselman AR, Watterberg KL. Analysis of neonatal clinical trials with twin births. BMC Med Res Methodol. 2009;9:12.

52. Sauzet O, Wright KC, Marston L, Brocklehurst P, Peacock JL. Modelling the hierarchical structure in datasets with very small clusters: a simulation study to explore the effect of the proportion of clusters when the outcome is continuous. Stat Med. 2012 Oct 1.

53. Dmitrienko A, Tamhane AC, Bretz F. Multiple Testing Problems in Pharmaceutical Statistics. Chapman & Hall/CRC; 2010.

54. Pellicer A, Valverde E, Elorza MD, Madero R, Gayá F, Quero J, et al. Cardiovascular support for low birth weight infants and cerebral hemodynamics: a randomized, blinded, clinical trial. Pediatrics. 2005 Jun;115(6):1501–12.

55. Pellicer A, Bravo MDC, Madero R, Salas S, Quero J, Cabañas F. Early systemic hypotension and vasopressor support in low birth weight infants: impact on neurodevelopment. Pediatrics. 2009 May;123(5):1369–76.

SafeBoosC phase II

56. Lundstrøm K, Pryds O, Greisen G. The haemodynamic effects of dopamine and volume expansion in sick preterm infants. Early Hum Dev. 2000 Feb;57(2):157–63.

57. Osborn D, Evans N, Kluckow M. Randomized trial of dobutamine versus dopamine in preterm infants with low systemic blood flow. The Journal of Pediatrics. 2002 Feb;140(2):183–91.

58. Trang TT, Tibballs J, Mercier JC, Beaufils F. Optimization of oxygen transport in mechanically ventilated newborns using oximetry and pulsed Doppler-derived cardiac output. Crit Care Med. 1988 Nov;16(11):1094–7.

59. Skinner JR, Boys RJ, Hunter S, Hey EN. Pulmonary and systemic arterial pressure in hyaline membrane disease. Arch Dis Child. 1992 Apr;67(4 Spec No):366–73.

60. Evans N, Kluckow M. Early determinants of right and left ventricular output in ventilated preterm infants. Arch Dis Child Fetal Neonatal Ed. 1996 Mar;74(2):F88–94.

61. Kluckow M, Evans N. Relationship between blood pressure and cardiac output in preterm infants requiring mechanical ventilation. The Journal of Pediatrics. 1996 Oct;129(4):506–12.

62. Osborn DA, Evans N, Kluckow M, Bowen JR, Rieger I. Low superior vena cava flow and effect of inotropes on neurodevelopment to 3 years in preterm infants. Pediatrics. 2007 Aug;120(2):372–80.

63. Osborn DA, Paradisis M, Evans N. The effect of inotropes on morbidity and mortality in preterm infants with low systemic or organ blood flow. Cochrane Database Syst Rev. 2007;(1):CD005090.

64. Takami T, Sunohara D, Kondo A, Mizukaki N, Suganami Y, Takei Y, et al. Changes in cerebral perfusion in extremely LBW infants during the first 72 h after birth. Pediatr Res. 2010 Nov;68(5):435–9.

65. Moran M, Miletin J, Pichova K, Dempsey EM. Cerebral tissue oxygenation index and superior vena cava blood flow in the very low birth weight infant. Acta Paediatr. 2009 Jan;98(1):43–6.

66. Kissack CM, Garr R, Wardle SP, Weindling AM. Cerebral fractional oxygen extraction in very low birth weight infants is high when there is low left ventricular output and hypocarbia but is unaffected by hypotension. Pediatr Res. 2004 Mar;55(3):400–5.

67. Kleinman CS, Seri I. Hemodynamics and Cardiology. 1st ed. Polin RA, editor. Saunders. Elsevier; 2008.

68. Jim W-T, Chiu N-C, Chen M-R, Hung H-Y, Kao H-A, Hsu C-H, et al. Cerebral hemodynamic change and intraventricular hemorrhage in very low birth weight infants with patent ductus arteriosus. Ultrasound Med Biol. 2005 Feb;31(2):197–202.

69. Wardle SP, Yoxall CW, Weindling AM. Determinants of cerebral fractional oxygen extraction using near infrared spectroscopy in preterm neonates. J Cereb Blood Flow Metab. 2000 Feb;20(2):272–9.

SafeBoosC phase II

70. Baenziger O, Stolkin F, Keel M, Siebenthal von K, Fauchere J-C, Kundu Das S, et al. The influence of the timing of cord clamping on postnatal cerebral oxygenation in preterm neonates: a randomized, controlled trial. Pediatrics. 2007 Mar;119(3):455–9.

71. Dani C, Pezzati M, Martelli E, Prussi C, Bertini G, Rubaltelli FF. Effect of blood transfusions on cerebral haemodynamics in preterm infants. Acta Paediatr. 2002 Jan 1;91(9):938–41.

72. Dani C, Pratesi S, Fontanelli G, Barp J, Bertini G. Blood transfusions increase cerebral, splanchnic, and renal oxygenation in anemic preterm infants. Transfusion. 2010 Jun;50(6):1220–6.

73. Schulze A, Whyte RK, Way RC, Sinclair JC. Effect of the arterial oxygenation level on cardiac output, oxygen extraction, and oxygen consumption in low birth weight infants receiving mechanical ventilation. The Journal of Pediatrics. 1995 May;126(5 Pt 1):777–84.

74. Cantagrel S, Cloarec S, Suc AL, Chamboux C, Tessier V, Saliba E, et al. Consequences of pulmonary inflations (sighs) on cerebral haemodynamics in neonates ventilated by high-frequency oscillation. Acta Paediatr. 1999 Sep;88(9):1004–8.

75. de Waal KA, Evans N, Osborn DA, Kluckow M. Cardiorespiratory effects of changes in end expiratory pressure in ventilated newborns. Arch Dis Child Fetal Neonatal Ed. 2007 Nov;92(6):F444–8.

76. Vanderhaegen J, Naulaers G, Vanhole C, de Smet D, van Huffel S,

Vanhaesebrouck S, et al. The effect of changes in tPCO2 on the fractional tissue oxygen extraction--as measured by near-infrared spectroscopy--in neonates during the first days of life. Eur. J. Paediatr. Neurol. 2009 Mar;13(2):128–34.

77. Dietz V, Wolf M, Keel M, Siebenthal von K, Baenziger O, Bucher H. CO2 reactivity of the cerebral hemoglobin concentration in healthy term newborns measured by near infrared spectrophotometry. Biol Neonate. 1999 Jan 1;75(2):85–90.

78. Pryds O, Greisen G, Skov LL, Friis-Hansen B. Carbon dioxide-related changes in cerebral blood volume and cerebral blood flow in mechanically ventilated preterm neonates: comparison of near infrared spectrophotometry and 133Xenon clearance. Pediatr Res. 1990 May;27(5):445–9.

79. Collins MP, Lorenz JM, Jetton JR, Paneth N. Hypocapnia and other ventilation-related risk factors for cerebral palsy in low birth weight infants. Pediatr Res. 2001 Dec;50(6):712–9.

80. Tin W, Milligan DW, Pennefather P, Hey E. Pulse oximetry, severe retinopathy, and outcome at one year in babies of less than 28 weeks gestation. Arch Dis Child Fetal Neonatal Ed. 2001 Mar;84(2):F106–10.

81. Chow LC, Wright KW, Sola A, CSMC Oxygen Administration Study Group. Can changes in clinical practice decrease the incidence of severe retinopathy of prematurity in very low birth weight infants? Pediatrics. 2003 Feb;111(2):339–45.

82. Anderson CG, Benitz WE, Madan A. Retinopathy of prematurity and pulse oximetry: a national survey of recent practices. Journal of perinatology : official journal of the California Perinatal Association. 2004 Mar;24(3):164–8.

SafeBoosC phase II

83. SUPPORT Study Group of the Eunice Kennedy Shriver NICHD Neonatal Research Network, Carlo WA, Finer NN, Walsh MC, Rich W, Gantz MG, et al. Target ranges of oxygen saturation in extremely preterm infants. N Engl J Med. 2010 May 27;362(21):1959–69.

84. Skov L, Pryds O. Capillary recruitment for preservation of cerebral glucose influx in hypoglycemic, preterm newborns: evidence for a glucose sensor? Pediatrics. 1992 Aug;90(2 Pt 1):193–5.

85. Pryds O, Christensen NJ, Friis-Hansen B. Increased cerebral blood flow and plasma epinephrine in hypoglycemic, preterm neonates. Pediatrics. 1990 Feb;85(2):172–6.

86. Harris RP, Helfand M, Woolf SH, Lohr KN, Mulrow CD, Teutsch SM, et al. Current methods of the US Preventive Services Task Force: a review of the process. Am J Prev Med. 2001 Apr;20(3 Suppl):21–35.

87. Woodward LJ, Anderson PJ, Austin NC, Howard K, Inder TE. Neonatal MRI to predict neurodevelopmental outcomes in preterm infants. N Engl J Med. 2006 Aug 17;355(7):685–94.
